# Fusion-based quantum computation

**DOI:** 10.1038/s41467-023-36493-1

**Published:** 2023-02-17

**Authors:** Sara Bartolucci, Patrick Birchall, Hector Bombín, Hugo Cable, Chris Dawson, Mercedes Gimeno-Segovia, Eric Johnston, Konrad Kieling, Naomi Nickerson, Mihir Pant, Fernando Pastawski, Terry Rudolph, Chris Sparrow

**Affiliations:** PsiQuantum, Palo Alto, 94304 CA USA

**Keywords:** Quantum information, Qubits

## Abstract

The standard primitives of quantum computing include deterministic unitary entangling gates, which are not natural operations in many systems including photonics. Here, we present fusion-based quantum computation, a model for fault tolerant quantum computing constructed from physical primitives readily accessible in photonic systems. These are entangling measurements, called fusions, which are performed on the qubits of small constant sized entangled resource states. Probabilistic photonic gates as well as errors are directly dealt with by the quantum error correction protocol. We show that this computational model can achieve a higher threshold than schemes reported in literature. We present a ballistic scheme which can tolerate a 10.4% probability of suffering photon loss in each fusion, which corresponds to a 2.7% probability of loss of each individual photon. The architecture is also highly modular and has reduced classical processing requirements compared to previous photonic quantum computing architectures.

## Abstract (web summary)

Fusion gates are common operations in photonic quantum information platforms, where they are employed to create entanglement. Here, the authors propose a quantum computation scheme where the same measurements used to generate entanglement can also be used to achieve fault-tolerance leading to an increased tolerance to errors.

## Introduction

In this paper we introduce fusion-based quantum computation (FBQC), a model of universal quantum computation that is built on two primitive operations: generation of small constant-sized entangled resource states and projective entangling measurements, which we refer to as fusion. In particular, we explore how topological fault-tolerant quantum computation for photonic architectures can be realized in this model.

All practical fault-tolerance schemes use measurements to reduce entropy. Circuit based implementations of the surface code use non-destructive four-qubit measurements to detect error syndromes. As the computation proceeds an extensive (in the size of the computation) amount of entanglement gets generated. One-way quantum computation^1^ achieves fault-tolerance using destructive single-qubit measurements on states containing an extensive amount of previously generated entanglement. Hence, these fault-tolerance schemes are both “measurement based”, although the terminology “measurement based quantum computing” (MBQC) is commonly associated with the latter. Fusion based quantum computation lies between these paradigms; it uses finite-sized entangled states and destructive entangling measurements to achieve fault-tolerance.

Measurements on photons can be extremely fast compared to matter-based systems, which coupled with their intrinsically low noise properties at first glance makes them ideal qubits. However measurement completely destroys the photons in the process. In order to circumvent this, many different schemes have been devised^1–9^, starting with the seminal result of Knill, Laflamme and Milburn^2^. These previous proposals for fault-tolerance in photonic architectures used entangling measurements to create the extensive entanglement required for one-way quantum computation followed by a separate set of single-qubit measurements for fault-tolerance. Fusion-based quantum computation combines these two stages, such that the same set of entangling measurements are used for creating extensive entanglement and for fault-tolerance leading to better performance.

**Fig. 1:**
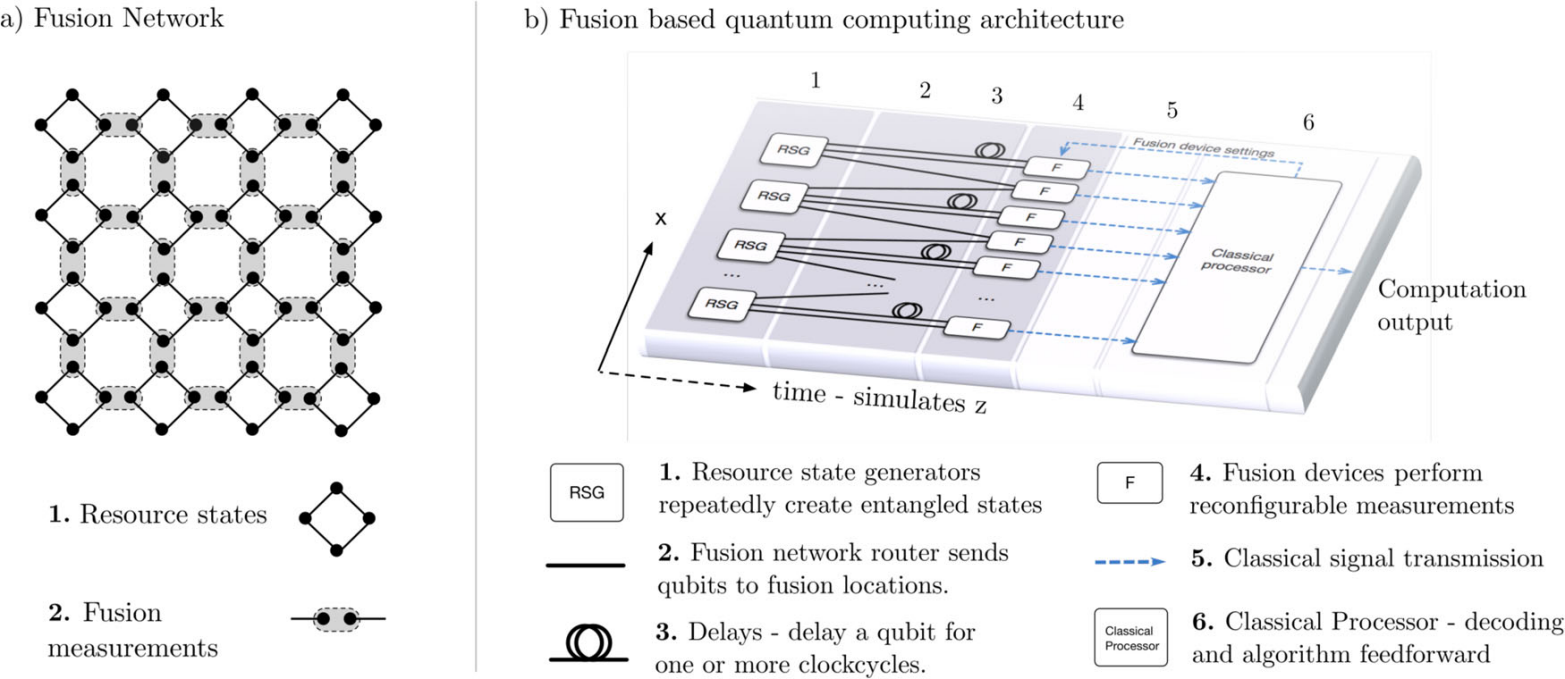
An example fusion network and schematic of a fusion based quantum computing architecture. **a** A 2D example of a fusion network, where entangled resource states and fusions are structured as a regular 2D square lattice. Resource states (1) are graph states made up of four entangled qubits in a square configuration. (2) These qubits are measured pairwise in entangling fusion measurements as depicted by the grey shaded ovals. **b** An example architecture which could create the fusion network shown in **a**. Each qubit is created in a resource state generator (1) and traverses the architecture from left to right through stages labelled 2–6. Qubits are connected to fusions (4) via a fusion network router (2), which can include time delays (3). Fusion devices may be reconfigurable such that they can make projective measurements in different bases. Classical signals from fusion measurements (5) are fed to a classical processor (6), which is responsible for decoding and algorithmic logic. There can be feedforward from this computation to reconfigure fusion measurements in order to implement logic. This figure illustrates how the fusion network may include one (or more) additional dimensions compared to the hardware. Here the fusion network is 2D and the physical hardware is a 1D array of resource state generation and fusion. The physical architecture for fault tolerant computing is discussed further in section 0.6.

The central principle of FBQC is to construct fusion networks from resource states and fusion measurements. The fusion network forms the fabric of the computation on which an algorithm can be implemented by modifying the basis of at least some of the fusion measurements. Appropriately combining fusion measurement outcomes gives the output of the computation. An example of a 2-dimensional fusion network is shown in Fig. 1a.

We have three main results. Firstly, we introduce FBQC as a computational paradigm and give a stabilizer formalism to evaluate its behavior. Secondly, we look at specific illustrative fusion networks and evaluate their performance. Thirdly, we define a physical architecture for FBQC in photonic systems. Even with the simple illustrative schemes in this paper, we see notable performance improvements over existing protocols^8,10,11^. We consider a hardware agnostic fusion error model and demonstrate a threshold of 11.98% against erasure in each fusion measurement, and a threshold of 1.07% against Pauli error. We consider a linear optical error model which accounts for photon loss and non-deterministic fusion operations. We present a scheme that can tolerate a 10.4% probability of suffering photon loss in each fusion, which corresponds to a 2.7% probability of loss of each individual photon. We also demonstrate a threshold of 43.2% against fusion failure with a ballistic scheme, compared to 14.9% previously reported^11^.

Construction of fusion networks requires physical components for generating resource states, performing fusion measurements and also for routing qubits and classical processing as shown in Fig. 1b. The schematic architecture in Fig. 1b illustrates one of the primary advantages of FBQC from a hardware perspective: the depth of physical operations is extremely low. Each qubit of a resource state is created, and then essentially immediately measured. This low depth is critical for minimizing accumulation of errors and tolerating leakage. Furthermore, in the examples of fault-tolerant fusion networks that we will present in this paper, it is sufficient for the fusion network routers to implement a fixed routing configuration. Fixed routing means that qubits produced from a given resource state generator will always be routed to the same location. This design feature is particularly appealing from a hardware perspective as it eliminates the need to be able to switch between multiple possible configurations, which may be error-prone (for example, integrated photonic components implementing fixed linear optical transformations are much higher fidelity that those that are reconfigurable), and reduces the burden of classical control. The resource state generation and fusion components shown in Fig. 1b can be implemented in any physical system. The focus of this paper is to describe how fault-tolerant quantum computation can be performed with these primitives. Efficient methods for performing resource state generation in photonic architectures are described in ref. ^12^ and designs of efficient switching networks are described in Bartolucci et al.^13^.

## Results and discussion

### Primitives of FBQC

1. Resource States: The first primitive of FBQC is a resource state which is a small entangled state. Resource states have a constant size and structure, regardless of the size of the computation they will implement. In this paper, we focus on qubit stabilizer resource states^14^, which can be described, up to local Clifford operations, by a graph *G* using the graph state representation^15^.
  Physically, this requires a system, called a resource state generator, that produces copies of the resource state to be consumed by the computation. This device can physically take many forms: it can produce photonic states or it can be a matter qubit device. The constant size of the resource state is crucial for fault tolerance, since it bounds errors on its qubits. Figure 2a shows an example of a resource state of six qubits that has the graph state representation of a ring of six.

**Fig. 2:**
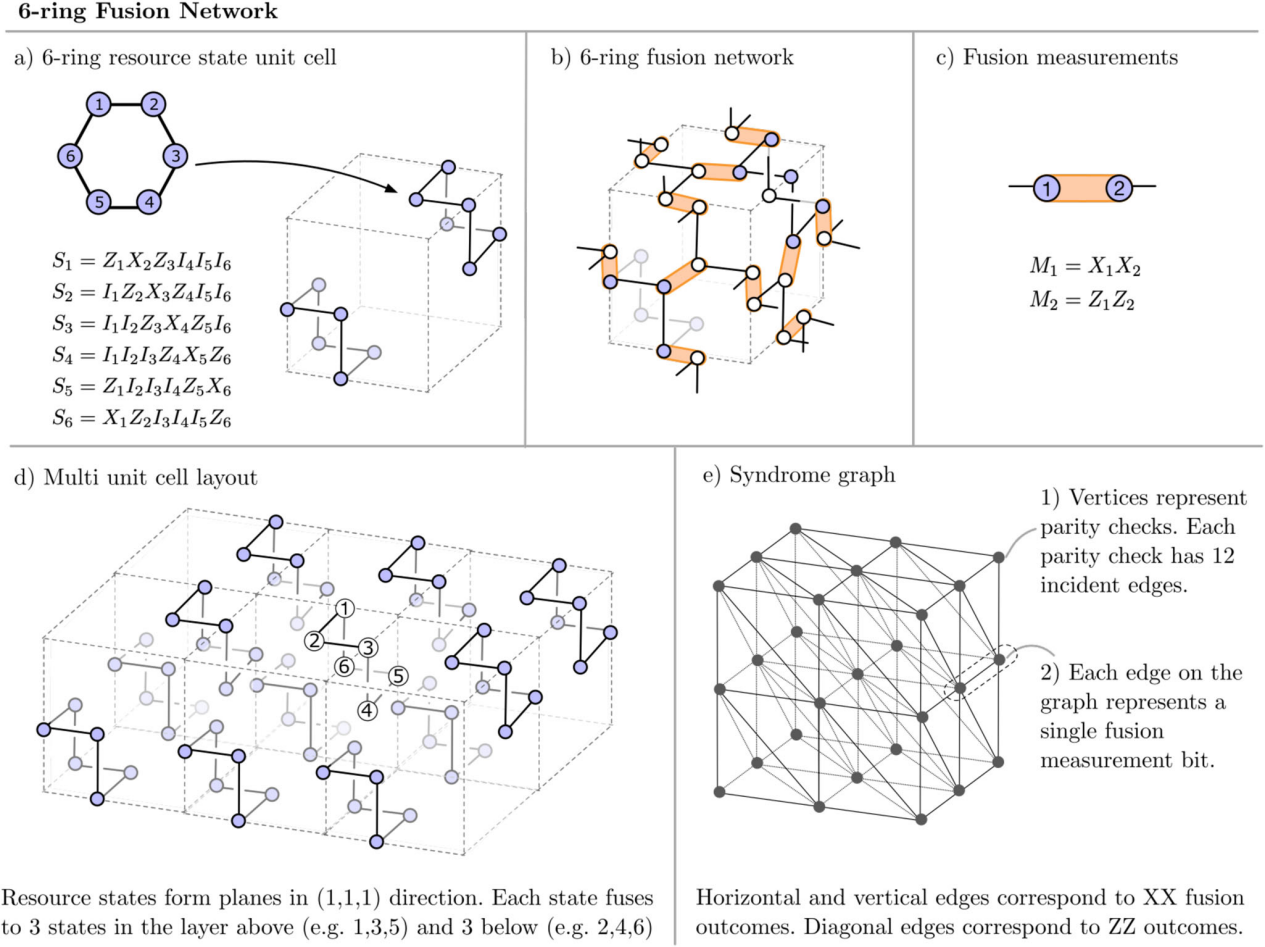
The “6-ring” fusion network. **a** Each resource state is a graph state in the form of a ring of 6 qubits. Two resource states are placed at opposite corners of each unit cell. **b** 2 qubit fusions connect every pair of qubits that share a face or an edge. Resource states that belong to the unit cell are shown as purple circles, while qubits from resource states in neighboring cells are shown as white circles. A formal definition of the fusion network can be found in Supplementary note VB. **c** All fusion measurements in the fusion network are two qubit projective measurements projective measurements on the bases *M*_1_ = *X*_1_*X*_2_ and *M*_2_ = *Z*_1_*Z*_2_. **d** Shows the layout of resource states across multiple unit cells. When unit cells are tiled, the resource states can be grouped into layers along 2D planes perpendicular to the (1,1,1) direction. Three qubits in each state fuse with the layer above, and three with the layer below. **e** The syndrome graph resulting from the fusion layout is a cubic graph with diagonal edges as shown. Primal and dual syndrome graphs have an identical structure. In both, the vertical edges correspond to *XX* type fusion outcomes and diagonal edges correspond to *ZZ* outcomes. The unit cells for primal and dual syndrome graphs can be interpreted as shifted by (1/2,1/2,1/2) so that each fusion corresponds to both a primal and dual edge which cross perpendicularly at the location of the fusion itself.
  A more detailed description of stabilizer resource states and their generation is presented in Supplementary note I and Bartolucci et al.^12^.
2. Fusion Measurement: The second primitive is a fusion measurement, a projective entangling measurement on multiple qubits. For example, a Bell measurement provides two output bits corresponding to Pauli observables *X*_1_*X*_2_ and *Z*_1_*Z*_2_ as shown in Fig. 2c. The physical implementation of a fusion device will depend on the underlying hardware. With dual-rail qubits, fusion can be performed by interferometric photon measurements between two resource states, which in its simplest form requires only beam splitters and photon detectors^3^. A more detailed description of fusion is presented in Supplementary note II.

### Fusion networks

The central objects in FBQC are fusion networks, which define a configuration of fusion measurements to be made on qubits of a collection of resource states. The fusion network forms the fabric of the computation on which algorithms are implemented by varying the basis of some of the fusion measurements. Appropriately combining fusion measurement outcomes gives the output of the computation. Figure 2b, d shows an example of a fusion network that uses the six-ring resource states and Bell measurements as fusions, both of which were described previously. We will refer to this as the “6-ring” fusion network.

In general there is no requirement for any particular structure in a fusion network, but since our goal here is to construct topologically fault-tolerant fusion networks, all the examples we look at are geometrically local.

To achieve fault tolerance, we must carefully choose resource states and fusion measurements in a fusion network such that the measurement outcomes combine to give parity checks of a fault tolerance scheme. We consider stabilizer fusion networks which can be characterized by two Pauli subgroups: (1) the resource state group *R* that is generated by the union of the stabilizers of all resource states in the fusion networks and (2) the fusion group, *F*, which is a Pauli sub-group that defines the fusion measurements. If fusions were perfect, we would learn the eigenvalues of all the operators in *F* by implementing the fusion network.

The key to fault tolerance is a redundancy between the Pauli operators measured during fusion, *F*, and the stabilizers of the resource states, *R*. This redundancy is reflected in the existence of a non-trivial check operator group *C* ≔ *R* ∩ *F*. The check group *C* can be interpreted as the subgroup of stabilizers *R* on the resource states which can be reconstructed by fusion measurements in *F*. In the absence of errors, the fusion measurement outcomes should be consistent with the resource state stabilizers. The outcomes form a (degenerate) linear binary code, which enables error correction. The stabilizer formalism by which we study error correcting code can be reformulated in this framework and we do this in Supplementary note IVA. Supplementary note IVB describes a simple example of a fusion network for which *R*, *F* and *C* can be explicitly written.

In the examples of topological fault tolerant fusion networks we study, all fusion outcomes are part of at least one check operator (generally two). Specifically, we will consider topological fusion networks that implement surface code-type fault-tolerance. The redundancy in these fault-tolerant fusion networks is well described by a syndrome graph representation. Every edge in the syndrome graph represents a binary measurement outcome, which determines the sign of a generator of *F*, and every node represents a generator of *C*. The parity of a check node in the syndrome graph is evaluated by taking the joint parity of all the adjacent edges. Given a set of fusion measurement outcomes each parity check has an associated parity value of either +1 or -1. The configuration of all of these parity outcomes is called the syndrome. If a fusion outcome is flipped, the vertices (checks) connected by its edge in the graph will have their parity values flipped. If a fusion outcome is erased or missing, the two checks connected by the edge in the graph can be multiplied/combined into a single check operator. The decoder uses values of the checks to infer the error class of the logical qubit. In the fault-tolerant fusion networks in this paper, the syndrome graph locally splits into two connected components that we refer to as primal and dual (there is no Poincare duality involved: the naming goes back to the syndrome graphs for error correction in the toric code). Each Bell fusion measurement contributes one outcome bit to each component. The syndrome graph representation allows existing decoders such as minimum-weight perfect matching and union-find decoders^16,17^ to be applied within the FBQC framework. Figure 2e shows the syndrome graph for the 6-ring fusion network, which is a cubic lattice with added diagonal edges. Each syndrome graph vertex has 12 incident edges. The primal and dual syndrome graphs for the 6-ring fusion network have an identical structure in the bulk. In Supplementary note VA, we present another fusion network called the 4-star fusion network, which we compare with the 6-ring fusion network in the next section.

### Error tolerance under hardware-agnostic error model

We quantify the fault-tolerant properties of the 6-ring fusion network by performing Monte carlo simulations under an error model, which we call the hardware-agnostic fusion error model, where every measurement outcome (i.e. every *XX* and every *ZZ* measurement from every fusion) is independently erased with probability ${p}_{{{{{{{{\rm{erasure}}}}}}}}}$ and flipped with probability *p*_error_. This allows capturing single qubit Pauli errors and erasures originating from resource state generation as well as those derived from the fusions measurements themselves. Compared to previous studies of fault-tolerant MBQC, which look at the erasure and error thresholds of single qubit measurements on lattices which already have long range entanglement^18,19^, this model captures errors in the joint measurements used to create long range entanglement starting from small resource states. Therefore, this error model is closer to a circuit level error model where individual resource states and fusion measurements play the role of elementary gates.

**Fig. 3:**
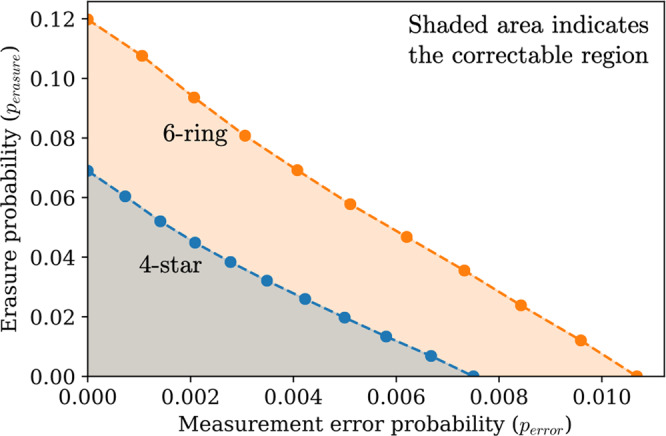
Performance of the six-ring (orange line) and 4-star (blue line, FBQC version of best architecture in literature^11^, see Supplementary note VA) fusion networks. The correctable region is shown for the two fusion networks under the two error parameters of the hardware-agnostic fusion error model: fusion erasure probability ${p}_{{{{{{{{\rm{erasure}}}}}}}}}$ and measurement error probability *p*_error_. Each marker shows the position of the threshold in the 2 parameter space, and is evaluated by a series of Montecarlo error sampling and decoding trials at different error parameters. Simulation details are provided in Supplementary note VII.

Setting ${p}_{{{{{{{{\rm{erasure}}}}}}}}}=(1-\beta )x$ and *p*_error_ = *βx*, we find the threshold value of *x* for different values of *β*. This allows us to map out a threshold curve which, for different ratios of the error parameters, gives the maximum values of *p*_error_ and ${p}_{{{{{{{{\rm{erasure}}}}}}}}}$ that can be simultaneously suppressed by making the fusion network larger. The full region where this suppression is possible is called the correctable region and is shaded in the figure. The fault tolerance thresholds were found using a minimum-weight perfect matching decoder^20,21^. The orange line in Fig. 3 shows the threshold curve for the 6-ring fusion network. The blue line in Fig. 3 is the threshold curve for a fusion network we term the “4-star fusion network”- an FBQC scheme for which the resource state is the four qubit Greenberger-Horne-Zeilinger (GHZ) state (see Supplementary note VA for details). The correctable region of the 4-star network is contained in the correctable region of the 6-ring network. The marginal ${p}_{{{{{{{{\rm{erasure}}}}}}}}}$ threshold for the 4-star network is 6.90%, while it is 11.98% for the 6-ring network. The marginal *p*_error_ threshold for the 6-ring network (1.07%) is also higher than for 4-star (0.75%).

### Loss tolerance for linear optical architecture

We now examine the performance of these fusion networks for a linear optical architecture. Here, we assume resource state generation to be ideal followed by independent loss on all photons. The fusions used in the fusion networks analyzed here attempt to measure *X*_1_*X*_2_ and *Z*_1_*Z*_2_ on the input qubits, which we label as 1 and 2 here. We will consider two types of imperfections in this linear optical error model: photon loss and the inherent probabilistic nature of linear optics. Both of these imperfections lead to erasure.

Fusion of dual-rail qubits with linear optics is intrinsically non-deterministic. In the absence of photon loss, a fusion operation will either herald ‘success’, indicating that *X*_1_*X*_2_ and *Z*_1_*Z*_2_ have been measured, or ‘failure’, in which case the fusion performs separable single qubit measurements. Depending on the linear optical circuit used to perform the fusion, the fusion can measure a pair of single qubit stabilizer measurement (e.g. *X*_1_ and *X*_2_, or *Z*_1_ and *Z*_2_) when it fails. It is simple to modify linear optical circuits to choose the failure basis using appropriate single qubit gates, which are easy to implement in linear optics, before a fusion. For instance, a fusion that measures *Z*_1_, *Z*_2_ on failure can be made to fail by measuring *X*_1_, *X*_2_ instead by placing a Hadamard gate before both input qubits. By taking the product of the two single qubit measurements we can reconstruct one of the intended two qubit measurements. Therefore, this event can be interpreted as a successful fusion with an erasure of one of the measurement outcomes. For example, if the intended fusion measurements were *X*_1_*X*_2_ and *Z*_1_*Z*_2_ and, upon fusion failure, we obtain single qubit measurement outcomes *X*_1_ and *X*_2_, we can treat this case as a successful fusion with an erased *Z*_1_*Z*_2_ measurement outcome. In this paper, the circuits used to implement fusion are randomized so that with 50% probability, the fusion measures *X*_1_,*X*_2_ on failure, and with 50% probability, *Z*_1_,*Z*_2_ are measured on failure. Since we’re using dual rail qubits which have a fixed number of photons (one), if one or more photons going into a fusion are lost, fewer than expected photons are detected and both *X*_1_*X*_2_ and *Z*_1_*Z*_2_ are erased, which we call the ‘erasure’ outcome.

We use two parameters in our linear optical error model. Every photon in the fusion is independently lost with probability *p*_loss_. If no photon in a fusion is lost, it succeeds with probability 1 − *p*_fail_ and fails with probability *p*_fail_.

A fusion between two dual-rail qubits inherently has a failure probability of 1/2. However, this probability can be reduced by “boosting” physical fusions using ancilliary entangled states^22^ in the fusion. However, boosting a fusion requires sending more photons to the fusion. We assume that photons from these ancillary entangled states have the same loss as the two photons from the two qubits being measured in the fusion. Therefore, boosting increases the probability of the erasure outcome in a fusion. We consider the family of fusion boosting protocols introduced by Grice^22^. For unboosted fusion *p*_fail_ = 1/2 and no ancilliary photons are required. If the fusion is boosted with a Bell pair, *p*_fail_ = 1/4 and there are two ancilliary photons in the fusion. In general^22^, *p*_fail_ = 1/2^*n*^ can be achieved by boosting a fusion with 2^*n*^ − 2 additional photons. For a fusion on *N* photons the probability that no photon in the fusion is lost is *η*^*N*^, where *η* = 1 − *p*_loss_. We therefore use a model of fusion erasure that captures the tradeoff between *p*_fail_ and the probability of losing a photon. The probability that no photon in the fusion is lost is ${\eta }^{1/{p}_{{{{{{{{\rm{fail}}}}}}}}}}$. Hence, with probability $1-{\eta }^{1/{p}_{{{{{{{{\rm{fail}}}}}}}}}}$, a fusion is erased. In this error model every individual physical fusion measurement in the network has an erasure probability of ${p}_{0}=1-(1-{p}_{{{{{{{{\rm{fail}}}}}}}}}/2){\eta }^{1/{p}_{{{{{{{{\rm{fail}}}}}}}}}}$, which we explain in detail in Supplementary note IIB.

The erasure probability due to both fusion failure and photon loss can also be reduced by using encoded fusion. This involves encoding every qubit in the resource state in a small code, and replacing each fusion in the network with an encoded fusion composed of transversal physical fusions. We consider encoding qubits in the (2,2) Shor code, which refers to a four qubit [[4,1,2]] quantum code which can be obtained by concatenating repetition codes for *X* and *Z* observables (see Supplementary note IIC for more details). With these techniques, the erasure from fusion failure is heavily suppressed and can be tolerated by both the 4-star and 6-ring networks. Encoded fusion requires a modification of resource states where every qubit in the original resource state is replaced by an encoded qubit, which in this case consists of four physical qubits. Figure 1 in Supplementary note I shows how the resource state for the 6-ring fusion network is modified with the (2,2) Shor encoding. The encoded resource state is a stabilizer state because the (2,2) Shor code is a stabilizer code.

**Fig. 4:**
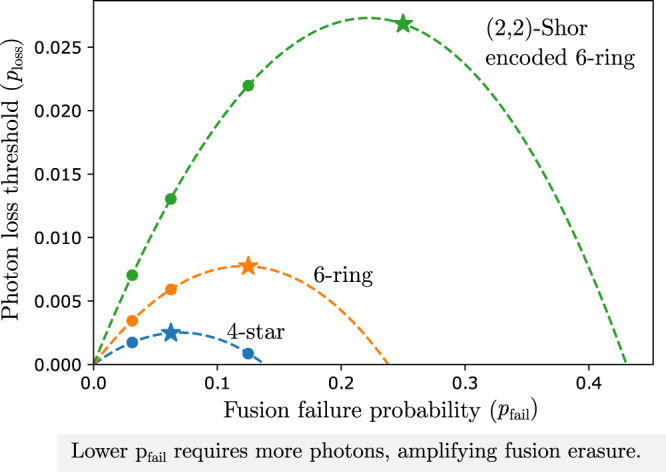
Photon loss threshold for the three fusion networks: 4-star (blue) and 6-ring (orange) and (2,2)-Shor encoded 6-ring (green). The threshold is calculated under the linear optical error model with the same photon loss probability *p*_loss_ applied to every photon in the protocol. We consider a physical model for fusion failure where *p*_fail_ = 1/2^*n*^ can be achieved by boosting a fusion with 2^*n*^ − 2 additional photons. Since more photons are required for these lower fusion failure rates, the effect of loss in this regime is amplified, with a probability ${(1-{p}_{{{{{{{{\rm{loss}}}}}}}}})}^{{2}^{n}}$ of no photon in the fusion being lost. Because of this the protocols demonstrate an optimal performance at some intermediate value of *p*_fail_. The markers represent the values of *p*_fail_ that can be achieved with fusions^22^ and the stars represent the optimum levels of boosting for the different schemes. The green curve corresponds to the 6-ring fusion network with qubits encoded in a (2,2)-Shor code. The details of the encoding and measurement scheme, and the error model used to evaluate these curves is explained in Supplementary note IIB and IIC.

We numerically model three fusion networks under this model of fusion and photon loss. The 4-star network, the 6-ring network, and an encoded 6-ring network. To evaluate the fusion networks in the linear optical error model we do not need to perform numerical simulations, but we can instead perform a mapping between *p*_fail_ and *p*_loss_, and the erasure parameter of our hardware-agnostic fusion error model: ${p}_{{{{{{{{\rm{erasure}}}}}}}}}$, and use the simulated threshold values from Fig. 3. In Fig. 4, we plot the threshold in photon loss of the fusion networks described above as a function of the fusion failure probability *p*_fail_. Although a low value of *p*_fail_, which is achieved by boosting the fusion, reduces erasure due to fusion failure, our model penalizes high levels of boosting by accounting for the loss on the increased number of boosting photons needed to achieve these low failure rates. As a result, there is an optimum value of *p*_fail_ for every fusion network which corresponds to an optimum level of boosting.

The blue and orange lines in Fig. 4 show the threshold behavior for the 4-star and 6-ring fusion networks respectively. The failure thresholds for these networks (in the absence of photon loss) is below 25%, which means that simple boosted fusion is not sufficient for fault tolerance. The markers represent the values of *p*_fail_ that can be achieved with fusions presented in^22^ and the stars represent the optimum levels of boosting. With the (2,2)-Shor encoding, the 6-ring fusion network provides a significantly larger marginal failure threshold of 43.2%. With the 25% failure probability achieved with fusions boosted with a Bell pair, we have a loss tolerance of 2.7% per photon. In other words, by boosting fusions with a Bell pair, the fusion network can be in the correctable region even when the probability of at least one photon being lost in a fusion is 10.4%.

### Quantum computation with fault-tolerant fusion networks

So far, we have only described how to create a fault tolerant bulk in FBQC - which behaves as the fabric of topological quantum computation. Creating the bulk is the most critical component of the architecture, as it is this that determines the error correction threshold. Fault-tolerant computation can be achieved simply by exchanging some fusions in the ‘bulk’ for single-qubit measurements, as discussed next.

**Fig. 5:**
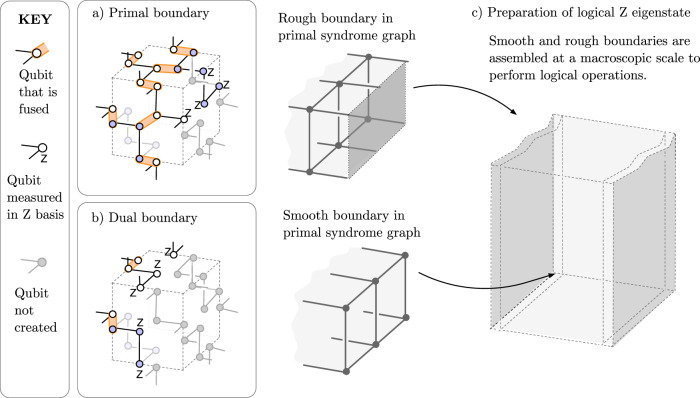
A scheme for creating boundaries that can be used to modify the bulk to perform quantum computation. Boundaries are classified as “primal” if they are “rough'' ("smooth'') for the primal (dual) syndrome graphs, and are classified as “dual” if they are “rough'' ("smooth'') for the dual (primal) syndrome graphs^16^. **a**, **b** show modified unit cells of a fusion network that can generate a primal and dual boundary respectively. In each, the fusion network is made up of the same configuration of resource states as in the bulk (see Fig. 2), but where some subset of the fusion measurements have been replaced with single qubit *Z* measurements, and some subset of the resource states are entirely removed (indicated by greyed out circles). If at the boundary a resource state has no remaining entangling operations connecting it into the bulk, then it does not need to be created. All the remaining fusions (shown by orange ovals) are a projective measurement on *XX*, and *ZZ*. The effect of this modified network is to truncate the bulk either at (**a**) a slice halfway through the cell or (**b**) at the edge of the cell. **c** shows an example of hows these unit cells can be composed to create macroscopic boundary conditions, enabling fault-tolerant logic. It corresponds to the initialization of a standard surface code in the computational basis.

Local modifications to the bulk are enough to implement Clifford gates fault-tolerantly. The locally modified bulk can be regarded as the space-time picture of a code with a topology that changes over time, i.e. that undergoes some form of (topological) code deformation^23,24^. The topological features involved might be boundaries^25^ or twists^26^, arranged in different manners^27–29^, but the common theme is that they can be implemented locally. In FBQC this can be achieved by modifying some of the fusion measurements, possibly to substitute them with single-qubit measurements. In Fig. 5, we show how boundaries can be created (using *Z* measurements), which is enough for lattice surgery techniques^27^. Twists are addressed in more detail by Bombín et al.^30^.

To achieve a universal gate set, the Clifford gates must be supplemented with state injection which, combined with magic state distillation protocols, can be used to implement *T* gates and other small angle rotation gates. Magic state injection can be implemented in FBQC by performing a modified fusion operation, by making a single qubit $\frac{\pi }{8}$ measurement, or by replacing a resource state with a special ‘magic’ resource state. Further details regarding implementation of logic can be found in Supplementary note VIA.

### Physical architecture

Fusion networks have no intrinsic notion of time or space, which allows a great amount of flexibility in how they are physically implemented. The fusion network does not specify the ordering of fusion measurements, nor is it necessary that all the resource states exist simultaneously. The same fusion network could be implemented by producing all the resource states simultaneously or by staggering the resource state generation such that only a portion of the fusion network is ‘alive’ at any given moment in time. Re-introducing physical space and time-ordering is an architectural design tool that can be used to adapt to the available hardware.

**Fig. 6:**
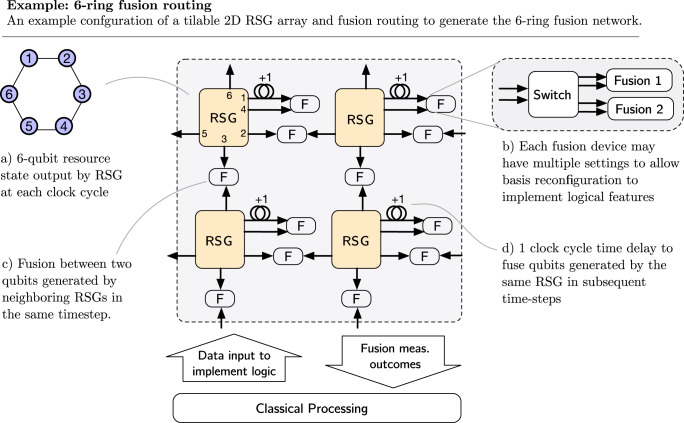
Example of a physical layout of resource state generators and fusion routing that can be used to create the 6-ring fusion network. **a** Four RSGs are shown, each producing a 6-ring state in each clock cycle. These are arranged in a tileable configuration. Qubits from each RSG are routed to 2-qubit fusion devices. **b** Each fusion device can include a switch that can reconfigure between multiple fusion settings to implement logical gates. Each RSG outputs 6 qubits per clock cycle. **c** Four qubits from each state immediately enter a fusion device in one of the four spatial directions: North, South, East or West. This generates entanglement between states created at neighboring sites in the same time step. **d** The two remaining qubits from each state are used to generate entanglement between states produced at the same physical location, but in different time steps. To achieve this, one qubit passes through a 1 clock cycle delay, so that it arrives at the fusion device coincidentally with a state produced in the following clock cycle. Fusion measurement outcomes are output from the system as classical data. In the bulk no data input is required, but classical control is needed at certain locations to reconfigure fusion devices to perform logical gates.

At the center of an architecture is the mechanism for generating resource states, which will be generated at a certain spatial location and a certain time. It is natural to consider the notion of resource state generators (RSGs), physical devices producing resource states at a certain clock speed. This picture is particularly relevant for photonic architectures. We can then consider the lifetime of a qubit, which is created in a resource state generator, passed into a fusion network router, which routes qubits to the right fusion location. The qubit is then destructively measured in a fusion. This very limited qubit lifetime is a strength of FBQC, particularly for photonic architectures where optical loss is the dominant source of physical error. In Fig. 6, we show an example configuration of a 2D array of RSGs connected to fusions by a fusion network router to implement the 6-ring example fusion network introduced in Fig 2.

The example in Fig. 6 demonstrates several desirable features of the physical implementation of a scheme for FBQC. The operational depth is extremely low and every qubit only needs to see a small constant depth of physical components in its lifetime, which is good for reducing loss and errors in the qubit lifetime. While a fusion network is composed of many resource state generators and fusion measurements, these components don’t need to co-exist simultaneously and the same physical components can be reused. In physical systems where the measurement time is much smaller than the time a qubit can be kept alive, a single RSG can be used to create an entire block of a fusion network by creating the network one resource state at a time^31^. This is particularly attractive in photonics where measurements can be performed on the sub-ns^32^ timescale and optical fiber can store photons for 5 μs with less than 5% loss^33^. Even when components are being reused, the routing required to implement the fusion network is fixed i.e. a resource state generated at a given physical location goes to the same fusion devices. This eases switching requirements in the architecture. The implementation of logic requires some fusion measurements to be reconfigurable as indicated in Fig. 6b. Boundaries or other topological features in the bulk are implemented by changing the measurement basis of the fusion or switching to single qubit measurements, but do not require re-routing of photons.

FBQC is a general framework for quantum computation that is built out of hardware primitives that are natural for many physical systems including photonics. We expect that this framework, with its ability to tightly link physical errors with their effect on quantum error correction, will allow performance improvements in systems that are fundamentally based on resource state generation and projective measurements, such as in linear optical quantum computing. Even with the simple examples we present here, we demonstrate a doubling of the threshold compared to previous schemes. As technology moves ever closer to realizing these systems, having such a theoretical framework will be an important tool for engineering hardware and architecture designs to achieve large scale fault-tolerant quantum computation.

### Tolerance to errors in FBQC

The thresholds presented in Section 0.3 are based on simple error models. In a physical implementation there will many physical sources of imperfection that contribute to the total erasure and measurement error rates, as well as the specific structure of those errors. A full analysis requires a detailed system architecture, and depends strongly on the specifics of physical error models. However the models we present here can still be used to get a meaningful insight into realistic performance. Since our model does not fix the ratio of Pauli and erasure errors, and since some correlated error structure is already present in the error model it is often possible that an exact or approximate analytical mapping can be made from a more detailed circuit-level error model to the numerical results we presented in Sec. 0.3. When it comes to specific structure in the errors, error bias and correlations impact the threshold, and time ordering of operations can spread errors. However, there is reason to believe that the impact of these can be limited in FBQC. In particular:

1. FBQC accounts for the structure of errors due to the creation of long range entanglement: As we build up large scale entanglement from low-weight physical operations, errors in resource states will lead to fusion measurement errors. The way errors propagate from resource state generation through fusion measurements is captured in the syndrome graph.
2. Resource state and fusion errors are intrinsically local: The construction of FBQC limits how far errors can potentially spread. Assuming they are created in physically separate systems we would expect correlations to exist only within a resource state and not between resource states (prior to fusion). This expectation is particularly strong with linear optics, where photons at different locations cannot become ‘accidentally’ entangled with one another since they do not interact. Furthermore, each qubit in the protocol has a short finite lifetime, limiting the potential for the spread of errors in its neighborhood.
3. Correlations within a fusion can only improve performance: A likely place for correlated errors to appear is between the two measurement outcomes of a fusion operation. Our model treats these errors as uncorrelated. Since we decode primal and dual syndrome graphs separately here, if fusion errors were correlated it would make no difference to our thresholds. If that information were to be accounted for in decoding it could only improve the performance.

Finally, when considering computation we need to account for the fact that logic gates are performed via creating topological features, such as boundaries or twists, which need different physical operations. We would therefore expect these to have different error models at those locations. It is, however, the case that in topological fault tolerance the bulk determines the threshold. The topological features used to implement logic are 2- or 1-dimensional objects. Our numerical results should therefore correctly indicate the threshold of fault-tolerant logic, although logic gates may have a different below threshold scaling behavior.

## Supplementary information

Supplementary Information

## Data Availability

The datasets generated during and/or analysed during the current study are available from the corresponding author on request.

## Code availability

Custom code used to generate data for the study will be made available to the interested reader upon request.
