# Supplementary material for: Fusion-based quantum computation
Source: Nat Commun. 2023 Feb 17;14:912. doi: 10.1038/s41467-023-36493-1 (PMC9938229; doi:10.1038/s41467-023-36493-1)
Supplement: Supplementary file 1 — Supplementary Information [file 41467_2023_36493_MOESM1_ESM.pdf]

Supplementary Information: Fusion-based quantum computation

Sara Bartolucci, Patrick Birchall, Hector Bombín*, Hugo Cable, Chris Dawson, Mercedes Gimeno-Segovia, Eric Johnston, Konrad Kieling, Naomi Nickerson *,† Mihir Pant *,‡ Fernando Pastawski, Terry Rudolph, and Chris Sparrow
PsiQuantum, Palo Alto
(Dated: February 15, 2023)

Supplementary Note I: Resource states

The small entangled states fueling the computation are referred to as resource states. Importantly, their size is independent of code distance used or the computation being performed. This allows them to be generated by a constant number of operations. Since the resource states will be immediately measured after they are created, the total depth of operations is also constant. As a result, errors in the resource states are bounded, which is important for fault-tolerance.

Here we focus on qubit stabilizer resource states [1], which can be described, up to local Clifford operations, by a graph G using the graph state representation [2]. The graph state is defined as the quantum state $|G\rangle$ obtained by putting qubits in the $|+\rangle$ state at each vertex and performing a controlled-Z gate between qubits for which the corresponding vertices in the graph are neighbors. Equivalently, n stabilizer generators for a graph state with vertices labeled from 1 to n are given by $X_i \prod_{j\in\mathcal{N}(i)} Z_j, i \in \{1, 2, ..., n\}$ where $\mathcal{N}(i)$ is the set of vertices neighboring vertex i in G.

Supplementary Fig. 1(a) shows an example of a resource state in the form of a 6-ring graph state, where the stabilizer generators for the state are $Z_6X_1Z_2$, $Z_1X_2Z_3$, $Z_2X_3Z_4$, $Z_3X_4Z_5$, $Z_4X_5Z_6$, $Z_5X_6Z_1$. This is one of the states we use in an example of a topological fault tolerant network in supplementary note V.

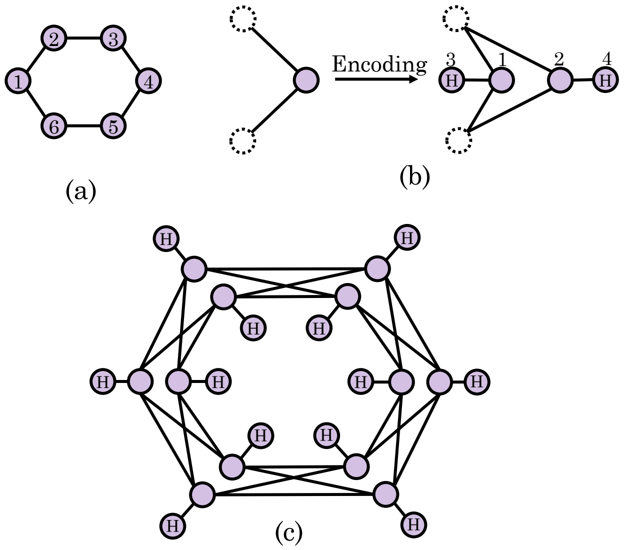

Supplementary Fig. 1: (a) An example of a resource state represented as a graph state. With the qubits labelled as in the figure, the stabilizers for the resource state are $Z_6X_1Z_2$, $Z_1X_2Z_3$, $Z_2X_3Z_4$, $Z_3X_4Z_5$, $Z_4X_5Z_6$ and $Z_5X_6Z_1$. (b) A qubit in the resource state can be replaced by a (2,2)-Shor encoded resource state with the depicted transformation. The qubits 1 and 2 both have the same neighboring qubits, drawn as dotted circles, as the unencoded qubit on the left. Qubits with an H inside have a Hadamard applied to them with respect to their graph state representation. (c) The resource state in (a) with every qubit encoded in a (2,2)-Shor code.

---

* these authors contributed equally
†Electronic address: naomi@psiquantum.com
‡Electronic address: mihir@psiquantum.com

We also consider *encoded resource states*, which can be used as a tool to reduce the impact of loss and errors in fusion measurements. In an encoded resource state the qubit operators (X_i, Z_i) are replaced by encoded qubit operators ($\bar{X}_i$, $\bar{Z}_i$). We consider the example of encoding each qubit in a graph state with the (2,2)-Shor code [3] (as defined in supplementary note II C), which has representative encoded operators:

$$\bar{X}_i = X_{i,1}X_{i,3} \tag{1}$$

$$\bar{Z}_i = Z_{i,1}Z_{i,2}, \tag{2}$$

and each encoded qubit has stabilizers

$$S = \langle X_{i,1}X_{i,2}X_{i,3}X_{i,4}, Z_{i,1}Z_{i,3}, Z_{i,2}Z_{i,4} \rangle. \tag{3}$$

This encoding is shown graphically in Supplementary Fig. 1(b), where the qubit is replaced by 4 qubits with appropriate graph state connections to result in the redundancy of the graph state stabilizers. Replacing every qubit of a 6-ring with a (2,2)-Shor encoded qubit gives us the resource state depicted in Supplementary Fig. 1(c). Supplementary note II C explains the structure and effect of an encoded fusion on these states in greater detail.

The operations used to create a resource state depend on the physical platform used, and it is worth noting that the quantum hardware used to implement fusions may differ from that used to create resource states. In solid state qubits, for example, resource states can be generated using unitary entangling gates[4–9] or dissipatively [10–12].

A. Resource state generation with linear optics

When using linear optics, generation of resource states is achieved by performing a series of projective measurements, such as fusions described in supplementary note II, on even smaller entangled states such as Bell states and 3-GHZ states which we sometimes refer to as *seed states*. Methods for the generation of seed states are fully covered in [13]. Since projective entangling measurements in linear optics succeed probabilistically, as will be discussed in the next supplementary note, it is often advantageous to use switching networks between fusions to enhance the success probability of the protocol. Using these networks, we attempt probabilistic operations multiple times and only select cases where they have succeeded. In this sense, multiplexing is used to effectively approximate post-selection on entangling fusion outcomes. Since the size and number of probabilistic operations required to generate a resource state is fixed, the resource overhead from repeating probabilistic operations is constant. There are many options for implementing such switching networks, depending on the required efficiency and available devices [14]. It is worth noting that to produce resource states that are qubit states, that is, states with multi-partite entanglement between well-defined qubits, the states at intermediate stages of the resource state generation do not themselves need to be qubits.

Any stabilizer state can be created using 3-GHZ states, Bell measurements and single-qubit Clifford unitaries. A trivial way to see this is that any graph state can be created by placing a d-GHZ state at every node of the graph, where d is the degree of the corresponding node, placing a qubit from each neighboring node along every edge in the graph and performing Bell measurement in the XZ, ZX basis along the edges. A d-GHZ can be created by performing Bell measurements in the XX, ZZ basis between qubits of 3-GHZ states. Finally, any stabilizer state is equivalent to a graph state upto single qubit cliffords [2]. This shows that any stabilizer resource state can be created in linear optics with 3-GHZ states, type-II fusions, clifford unitaries and multiplexing. However, there are more efficient methods for creating resource states, discussed in [13].

Determining the most suitable resource state is part of the design of an FBQC scheme for a realistic hardware implementation, as the noise profile of the resource state will depend on the generation protocol used. For a given target resource state there is an enormous number of possible preparation protocols, each of which will result in a different noise profile. However, the fixed size of the resource state implies that any generation protocol will require a finite number of operations, and therefore the noise accumulated in any of the state generations will be bounded. Moreover, any error correlations that emerge from independent state generation will be local to that state, which limits the spread of errors in the fusion network and is discussed in the subsection "Tolerance to Errors in FBQC" in the section on "Quantum Computation with Fault-Tolerant Fusion Networks" in the main paper.

Supplementary Note II: Fusion

In FBQC the initial quantum resources are small entangled resource states of a fixed size. The large scale quantum correlations necessary for universal computation are generated when we perform measurements on qubits from distinct resource states. In order for this to generate long range entanglement at least some of the measurement outcomes need to be entangling, i.e. projectors onto a subspace containing at least one entangled state. There is a long history of work [15–19] exploring different types

of multi-qubit measurements as a computational primitive. Here, differently to these previous works, we focus on (potentially destructive) projective measurements with only rank 1 outcomes.

In general, the measurement could be any positive operator valued measure (POVM) but, for the purposes of achieving fault tolerance, it is helpful to consider measurements where all outcomes are projections onto stabilizer states. This makes it possible to use existing stabilizer fault tolerance methods. In the examples in this paper we focus on the case of two-qubit measurements which are Bell state projections, and we follow [20] in calling this *Bell fusion*. Using Pauli operator notation we can describe a Bell fusion as measuring the operators X_1X_2, Z_1Z_2 on the two input qubits, where X_i (Z_i) is the single qubit Pauli-X (Z) operator on the qubit i. This measurement is rank 1, and the measurement operators form a stabilizer group. We will sometimes refer to this as an $\langle X_iX_j, Z_iZ_j\rangle$ fusion, where we are using the notation $\langle s_1, s_2\rangle$ to indicate a group generated by the operators s_1, s_2.

In the fusion networks we study here, the vast majority of fusion measurements needed to implement quantum error correction are identical Bell fusions. However, in order to implement logical gates some fraction of the measurements need to differ from the others. This can be by modifying Bell fusions to implement different two-qubit stabilizer measurements or introducing single qubit measurements.

A. Fusion in Linear Optics

In linear optical quantum computing (LOQC), Bell fusion on pairs of photonic qubits is simple to perform, but does not deterministically generate entanglement. This non-determinism means that the desired two-qubit measurement outcomes are sometimes exchanged for single-qubit stabilizer measurements, an event we refer to as *fusion failure*. Architectures for LOQC must handle these non-determinstic operations. In the FBQC schemes we describe here, these fusion failures are directly dealt with by the quantum error correction protocol.

Here we specifically consider 'dual-rail' qubits composed of a single photon in two photonic modes. A photon in the first mode represents the state $|0\rangle$ and a photon in the other mode represents $|1\rangle$. This qubit encoding is attractive because it contains a fixed number of photons and so loss takes the qubit out of the computational subspace. When the qubit is measured photon detectors will count the total number of photons, and therefore a loss can be heralded, which is a powerful tool for being able to tolerate optical loss. Another advantage of the fact that all qubit states have a definite photon number in dual-rail encoding is that relative phase shifts between the optical modes of different qubits have no effect on the qubit state. This arbitrary tolerance to inter-qubit phase errors is key to the ability to store qubits in longer delay lines as shown in Fig. 5 in the main paper and discussed in more length in [21]. Note that the relative phase between the two modes that make up a single dual-rail qubit must still be carefully preserved but this local control is much easier to achieve than for qubit encodings in which phase shifts between qubits can implement non-trivial operations. In the dual-rail encoding all single qubit operations can be implemented deterministically and with high accuracy with linear optical operations [22]. The single qubit operations we make use of here are a Hadamard operation which corresponds to a 50/50 beamsplitter and a phase gate which corresponds to a phase shift on one rail of the qubit.

Bell fusion on dual-rail qubits can be implemented using a linear optical circuit in which all four modes of the two qubits are measured. This is often referred to as type-II fusion [20]. This fusion has three possible outcomes, which are shown in Supplementary Fig. 2. Fusion can 'succeed' which happens with probability $1 - p_{\text{fail}}$. In this case the input qubits are measured in the Bell stabilizer basis X_1X_2, Z_1Z_2 as intended.

The fusion 'fails' with probability p_{fail}, in which case it performs separable single qubit measurements Z_1I_2, I_1Z_2[1]. In case of such a failure, one of the two desired outcomes, Z_1Z_2, can still be obtained by multiplying the two single qubit measurements together. Therefore fusion failure can be treated as a Bell measurement where one of the two measurement outcomes is erased. Note that this does not generate entanglement, rather it is making use of the available classical information. In the presence of hardware imperfections (leading to photon loss and other physical level errors), there is a third possible outcome: fusion "erasure". In this case neither of the intended stabilizer outcomes is measured.

When using dual-rail qubits with path encoding[2], the simplest way to implement a type-II fusion involves only two beam splitters and four detectors, and has a failure probability $p_{\text{fail}} = 50\%$ [20]. Using an additional Bell pair, fusion can be 'boosted' to suppress the failure probability to 25% [23, 24], and by using more ancillary photons the fusion success rate can be boosted further. It is important to note that fusion failure is a more benign error than erasure, it is not only heralded, but failed fusions produce *pure* quantum states that can still be used in the computation without adding any additional noise.

---

[1] In [20] the failure basis is X_1X_2, changing the failure basis is achieved by a simple reconfiguration of the physical device

[2] In the dual-rail encoding, a qubit is represented by a single photon being in either of two modes. When using path encoding, the two modes correspond to two distinct waveguides, $|0\rangle$ corresponds to the photon being in the first waveguide and $|1\rangle$ to the photon in the second waveguide.

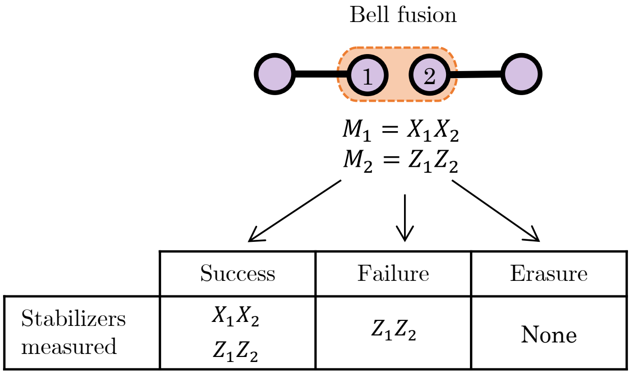

Supplementary Fig. 2: Outcomes of a linear optical Bell fusion. A fusion on qubits from two cluster states is shown, with intended outcomes X_1X_2 and Z_1Z_2. In the presence of photon loss there are three possible outcomes: *fusion success* where both measurement outcomes are obtained, *fusion failure* where only the outcome Z_1Z_2 is obtained, and *fusion erasure* where no measurement outcome is obtained. Fusion failure is intrinsic in a linear optical implementation, and happens even when all operations are ideal. Fusion erasure only occurs due to errors in the system, most commonly if one or more of the photons going into the fusion measurement are lost.

B. Linear optical fusion error model

A fusion attempts to measure two input qubits 1 and 2 in the Bell basis X_1X_2, Z_1Z_2. However linear optical fusion on two dual-rail qubits does not always perform these measurements because of the inherent non-determinism of dual-rail entanglement generation with linear optics and the presence of photon loss. In the example in supplementary note V, we primarily consider linear optical Bell fusion where every photon, including the photons used in boosting, is lost with the same probability p_{loss} and $\eta := 1 - p_{\text{loss}}$. In the linear optical error model, we assume that the probability of a fusion having no lost photon is $\eta^{1/p_{\text{fail}}}$ where p_{fail} is the probability of the fusion failing. This model accounts for the fact that boosting to obtain lower fusion failure probabilities involves using a larger number of photons as ancillas in the fusion, which implies a higher risk of fusion erasure due to photon loss. If the fusion is unboosted, $p_{\text{fail}} = 1/2$, there are only two photons in the fusion and the probability that no photon in the fusion is lost is η^2. If the fusion is boosted with a Bell pair [23], p_{fail} = 1/4, there are four photons in the fusion (two input photons and two photons from the ancilla Bell pair) and the probability that no photon in the fusion is lost is η^4. In general, [23] shows that $p_{\text{fail}} = 1/2^n$ can be achieved by boosting a fusion with $2^n - 2$ additional photons, resulting in a probability η^{2^n} of no photon in the fusion being lost. If any photon in a fusion is lost, fewer than expected photons are detected and both fusion outcomes from the fusion are erased.

When all photons are detected in the fusion, a fusion fails with probability p_{fail}, and instead of the intended measurements of X_1X_2 and Z_1Z_2, the fusion performs separable single qubit measurements. Depending on the linear optical circuit used to perform the fusion, the fusion can measure a pair of single qubit stabilizer measurement (e.g. X_1 and X_2 or Z_1 and Z_2) when it fails[3]. By taking the product of these two single qubit measurements we can reconstruct the two qubit measurement, therefore this event can be interpreted as a successful fusion with an erasure of one of the measurement outcomes. For example, if the intended fusion measurements were X_1X_2 and Z_1Z_2 and, upon fusion failure, we obtain single qubit measurement outcomes X_1 and X_2, we can treat this case as a successful fusion with an erased Z_1Z_2 measurement outcome

In this paper, the circuits used to implement fusion are randomized so that with 50% probability, the fusion measures X_1, X_2 on failure, and with 50% probability, Z_1, Z_2 are measured on failure. The probability that no photon in the fusion is lost is $\eta^{1/p_{\text{fail}}}$ and the erasure probability for a fusion measurement in the absence of loss is $p_{\text{fail}}/2$ in this randomized model. Consequently, the erasure probability for both the X_1X_2 and Z_1Z_2 measurements coming from fusion failure is $p_{\text{fail}}\eta^{1/p_{\text{fail}}}/2$. The probability of measuring both X_1X_2 and Z_1Z_2 is $(1 - p_{\text{fail}})\eta^{1/p_{\text{fail}}}$. With this randomization of the failure basis, the marginal probability of erasure for individual measurements coming from the fusion is

$$p_0 = 1 - (1 - p_{\text{fail}}/2)\eta^{1/p_{\text{fail}}}, \tag{4}$$

[3] It is simple to modify linear optical circuits to choose the failure basis using appropriate single qubit gates, which are easy to implement in linear optics, before a fusion. For instance a fusion that measures Z_1, Z_2 on failure can be made to fail by measuring X_1, X_2 instead by placing a Hadamard gate before both input qubits.

which we call the physical fusion measurement erasure probability. Since the two measurements from a fusion go to different syndrome graphs (primal and dual) that are decoded separately, evaluating the fault-tolerance threshold only requires this marginal erasure probability. Supplementary table I summarizes the probability of obtaining different measurements in a fusion boosted by a Bell pair with $p_{\text{fail}} = 1/4$.

Fusion outcome	success	failure in X	failure in Z	no-info
Probability	$3\eta^4/4$	$\eta^4/8$	$\eta^4/8$	$1-\eta^4$
Stabilizers measured	X_1X_2, Z_1Z_2	X_1, X_2	Z_1, Z_2	None

Supplementary Table I: Probability of measuring different stabilizers when performing a linear optical fusion boosted with a Bell pair ($p_{\text{fail}} = 1/4$) that attempts to measure X_1X_2, Z_1Z_2 and failure basis randomly chosen between X_1, X_2 and Z_1, Z_2. p_{loss} is the loss seen by every photon, including boosting photons, and $\eta = 1 - p_{\text{loss}}$.

By placing a Hadamard gate before one of the input qubits going into the fusion, we obtain a fusion that measures X_1Z_2 and Z_1X_2 with both measurements having the same marginal erasure probability of $1-(1-p_{\text{fail}}/2)\eta^{1/p_{\text{fail}}}$. This fusion measurement basis is used in the star fusion network in supplementary note V and the example fusion network in supplementary note III.

C. Encoded fusion erasure probability

In order to increase loss tolerance, the qubits in a resource state are encoded in a four qubit (2,2)-Shor code in supplementary note V, which is a $[[4,1,2]]$ CSS code obtained from the concatenation of two 2-qubit repetition codes. The (2,2)-Shor code can be oriented in two ways: with code stabilizers $\langle X_1X_2X_3X_4, Z_1Z_3, Z_2Z_4\rangle$ (X repetition above Z) or $\langle Z_1Z_2Z_3Z_4, X_1X_3, X_2X_4\rangle$ (Z repetition above X).

We perform fusion between two encoded qubits, which we label as A and B, by performing pairwise fusions transversally. Considering the case of X repetition above Z, the Shor code stabilizers are $\langle X_{1A}X_{2A}X_{3A}X_{4A}, Z_{1A}Z_{3A}, Z_{2A}Z_{4A}, X_{1B}X_{2B}X_{3B}X_{4B},$ $Z_{1B}Z_{3B}, Z_{2B}Z_{4B}\rangle$. The logical operators for the Shor encoded qubits are $\overline{X_A} = X_{1A}X_{3A}$ and $\overline{Z_A} = Z_{1A}Z_{2A}$, and the same for the B side. The encoded fusion measurements we would like to perform are $\overline{X_AX_B}$ and $\overline{Z_AZ_B}$. The physical measurements are $X_{iA}X_{iB}, Z_{iA}Z_{iB}, i \in \{1,2,3,4\}$.

Multiplying with the code stabilizers gives us multiple ways to reconstruct the encoded XX measurement from the physical fusion measurements:

$$\overline{X_AX_B} = (X_{1A}X_{1B})(X_{3A}X_{3B}) = (X_{2A}X_{2B})(X_{4A}X_{4B})$$

where the terms inside the brackets are fusion measurements. There are two ways to measure $\overline{X_AX_B}$ and each requires two physical fusion measurements. Therefore, the erasure probability of $\overline{X_AX_B}$ is $[1-(1-p_0)^2]^2$, where p_0 is the physical fusion measurement erasure probability.

Similarly,

$$\begin{aligned}\overline{Z_AZ_B} &= (Z_{1A}Z_{1B})(Z_{2A}Z_{2B}) = (Z_{1A}Z_{1B})(Z_{4A}Z_{4B}) \\ &= (Z_{2A}Z_{2B})(Z_{3A}Z_{3B}) = (Z_{3A}Z_{3B})(Z_{4A}Z_{4B})\end{aligned}$$

i.e. measuring $\overline{Z_AZ_B}$ requires one measurement from $\{(Z_{1A}Z_{1B}), (Z_{3A}Z_{3B})\}$ and one measurement from $\{(Z_{2A}Z_{2B}), (Z_{4A}Z_{4B})\}$. Therefore, the erasure probability of $\overline{Z_AZ_B}$ is $1-(1-p_0^2)^2$.

If the orientation of the Shor code is changed, i.e. we have Z repetition above X, the expressions for the encoded XX and ZZ erasure probabilities are flipped. In this paper, we randomly choose the orientation of the Shor code so that the probability of erasure for $\overline{X_AX_B}$ and $\overline{Z_AZ_B}$ are both

$$p_{\text{enc}} = \frac{[1-(1-p_0)^2]^2 + 1 - (1-p_0^2)^2}{2} \tag{5}$$

When $\eta = 1$ and $p_{\text{fail}} = 1/4$ as is the case for boosted fusion, $p_0 = 1/8$. This gives us $p_{\text{enc}} = 0.043$ which is the baseline erasure probability. $p_{\text{enc}} < p_0$ when $p_0 < 0.5$.

(a)

(b)

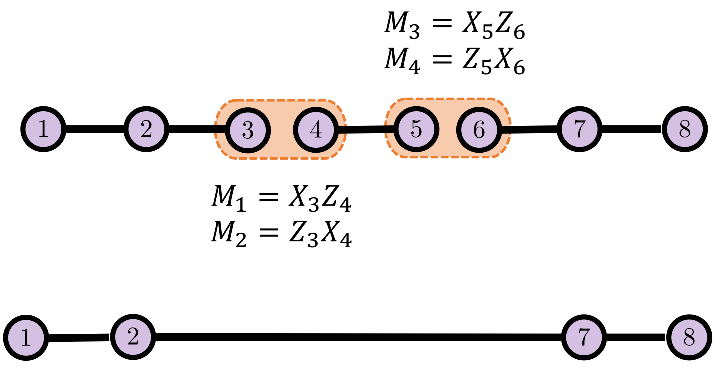

Supplementary Fig. 3: (a) An example of a fusion network with three resource states: a two qubit graph state, and two copies of a 3-qubit linear graph state. There are two fusions, shown by the orange lines, both of which measure the operators $\langle XZ, ZX\rangle$. Specifically, the resource state composed of qubits $\{1, 2, 3\}$ is stabilized by $\langle Z_1X_2Z_3, X_1Z_2I_3, I_1Z_2X_3\rangle$ and similarly for $\{6, 7, 8\}$. The qubits $\{4, 5\}$ are stabilized by $\langle X_4Z_5, Z_4X_5\rangle$. If every measurement result in the fusion network is successful and returns a $+1$ eigenvalue, the unmeasured qubits $\{1, 2, 7, 8\}$ are stabilized by $\langle X_1Z_2, Z_1X_2Z_7, Z_2X_7Z_8, Z_7X_8\rangle$ which corresponds to the 4-line graph state shown in (b).

Supplementary Note III: Fusion networks

A fusion network specifies an arrangement of resource states and a set of fusion measurements to be made on qubits of the resource states. After the measurements are made the qubits that were fused are removed from the state, and we learn measurement outcomes from each fusion. Two types of information remain: classical information from the measurement outcomes, and (potentially) some quantum correlations corresponding to unmeasured qubits. These measurement outcomes contain correlations that are the outcome of the fusion network, giving us both a computational output or, in the case of fault-tolerant fusion networks, parity checks that can be used for error correction. In this supplementary note, we describe how to construct fusion networks, and how to analyze them to identify the quantum and classical correlations that exist after fusion measurements have been made. In particular, we focus on *stabilizer* fusion networks where resource states are stabilizer states and fusion measurements are stabilizer projections.

Stabilizer fusion networks can be characterized by two Pauli subgroups: (1) A stabilizer group, R, describing the ideal resource states and (2) the fusion group, F, which is a Pauli sub-group that defines the fusion measurements, where we include $-\mathbf{1} \in F$.

F is not a stabilizer group since the signs of the operators are determined only after measurement (when an element of F anti-commutes with an element of R the outcome of the fusion measurements will be random). It is therefore convenient to include $-\mathbf{1}$ in our definition of F.[4]

The result of the fusion process, in particular the relationship between the measurement outcomes and the final state of the remaining qubits, can be understood using the stabilizer formalism. As it turns out a key role is played by the *surviving stabilizer group*, which is the set of elements of R that commute with all elements of F. This is also known as the centralizer of F in R, for which we use the following notation:

$$S := \mathcal{Z}_R(F), \tag{6}$$

Since in general F and R do not commute, the stabilizers after measurement will be updated. If fusion measurements are non-destructive, the new stabilizer of the final state will include the surviving stabilizer group, S. For an ideal fusion process, which *is* destructive, the state of the *outer qubits* (those remaining after measurement) is instead described by the stabilizer S_{out}, which is the restriction of S to the outer qubits up to signs. The signs are determined by the fusion measurement outcomes, and the elements of S are a handy guide for determining these signs. Namely, if $s_{\text{in}} \otimes s_{\text{out}} \in S$, where 'in' refers to the inner (or measured) qubits, then the eigenvalue $m = \pm 1$ of s_{in} is determined by fusion outcomes and we have:

$$m s_{\text{out}} \in S_{\text{out}}. \tag{7}$$

---

[4] Defining F to include $-\mathbf{1}$ is also convenient when defining the check group in supplementary note IV because a product of fusion measurements that is equal to an element of the resource state group *up to signs* gives us a check.

In other words, the surviving stabilizer group both determines the correlations on the post-fusion state and describes the origin of those correlations.

A simple example of a fusion network that creates a larger resource state from several smaller states is shown in Supplementary Fig. 3(a). The resource state group is generated by the union of the stabilizers of different resource states that can be inferred from their graph state representation:

$$\begin{aligned} R = \langle (X_1Z_2, Z_1X_2Z_3, Z_2X_3), (X_4Z_5, Z_4X_5), \\ (X_6Z_7, Z_6X_7Z_8, Z_7X_8)\rangle, \end{aligned} \tag{8}$$

where the brackets indicate the stabilizer generators associated with a single resource state. The fusion group is generated by the union of all fusion measurement operators,

$$F = \langle (X_3Z_4, Z_3X_4), (X_5Z_6, Z_5X_6), -\mathbf{1}\rangle, \tag{9}$$

where the brackets indicate measurement operators from the same fusion. The classical information produced by the fusion network comes from the measurement results of F. We use m_i to denote the classical measurement outcome of the measurement operators labelled M_i as shown in Supplementary Fig. 3(a). After fusion measurements are made, the output stabilizer generators, S_{out}, are given by:

$$S_1 = X_1Z_2, \tag{10}$$
$$S_2 = m_2m_4Z_1X_2Z_7, \tag{11}$$
$$S_3 = m_1m_3Z_2X_7Z_8, \tag{12}$$
$$S_4 = Z_7X_8, \tag{13}$$

where the signs of S_2 and S_3 are determined by the fusion measurement outcomes, $m_1 - m_4$. The output of this fusion network therefore corresponds to the 4 qubit linear graph state shown in Supplementary Fig. 3(b). By starting with resource states and fusions we are left with quantum correlations on the remaining qubits, with signs that are determined by measurement outcomes from the fusions in the network. This is the same principle that we will deploy to achieve fault tolerance in the next supplementary note.

It is worth noting that a fusion network as we have described it here simply represents the entanglement structure of states and measurements. It does not specify the ordering of operations, nor is it necessary that the entire fusion network exists simultaneously. When considering an architecture to implement a fusion network a specific time ordering can be introduced as a tool for architectural design, which is explored further in [21].

In the next supplementary note, we describe how redundancy can be added to a fusion network for fault-tolerance, and in supplementary note V we present several explicit examples of fault-tolerant fusion networks.

Supplementary Note IV: Fault-tolerant fusion networks

Fusion networks can be constructed to be fault tolerant, such that errors in resource states, or noisy fusion measurements can be corrected for, as long as errors occur with sufficiently low probability. In this supplementary note we describe fault tolerance in fusion networks.

Fault-tolerant fusion networks (FTFNs) can be constructed in a way that is inspired by circuit-based quantum error correction or fault-tolerant cluster states. This approach can be a useful initial guide, but a direct translation often yields inefficient schemes, and better approaches can be found by working more directly in the fusion network picture, as we show in the examples in supplementary note V. Here we consider the stabilizer structure of fusion networks in a fault tolerant setting, which build on the formalisms introduced for fault tolerant cluster states first introduced by Raussendorf et al. in [25] and extended in [26].

A. Stabilizer formalism for FTFNs

The surviving stabilizer group helps us describe the output of a fusion network, notably through two of its subgroups: the output stabilizers and the check operators.

Output stabilizers. The output stabilizers, S_{out}, can be thought of as the logical operators of the system. In a fault-tolerant fusion network the existence of check operators allows errors in measurements to be identified and corrected, adding redundancy to the output stabilizers.

Check operators. The key to fault tolerance is a redundancy in the fusion outcomes, which is precisely captured by the check operator group,

$$C := R \cap F. \tag{14}$$

The elements of this stabilizer group are the stabilizers of R that are effectively measured in the fusion process.

In the absence of errors fusion outcomes are such that the generators of the check group, C, have positive eigenvalues, even though the individual fusion outcomes are random. That is, the set of all possible fusion outcomes forms a binary classical code. Recall that $-\mathbf{1} \in F$ so elements of F and R that are equal up to signs will still result in checks.

Errors When imperfections are present resource states will differ from the ideal state, resulting in Pauli errors on some qubits. The fusion measurements may also be imperfect, adding additional noise, or leading to flipped measurement outcomes. In considering these different sources it is helpful to describe all errors as an element of the Pauli group applied to the qubits in the fusion network after ideal resource state preparation, and before ideal fusion measurements. This is sufficient to capture both imperfections in resource generation and errors that occur during fusion.

Detectable Errors Detectable errors are those Pauli operators that affect one or more of the check operators, such that their measured eigenvalue is negative. If an error, E, anti-commutes with a fusion measurement then the value of the fusion measurement will be flipped. The value of all check operators is referred to as the *syndrome*. The classical fusion outcomes form a classical linear binary code, which allows them to be corrected. Given the syndrome, it is the job of a decoder to predict the sign of the output stabilizer based on the most likely physical error class consistent with the detection pattern. If the decoder predicts the error class correctly, all logical outcomes will be recovered correctly. Note that the error correction being performed here is entirely on the classical fusion measurement outcomes, and the output of decoding is used to update the Pauli frame that allows us to interpret the remaining state. There is no notion of a physical correction that could be applied, since the qubits that suffered errors have been destructively measured.

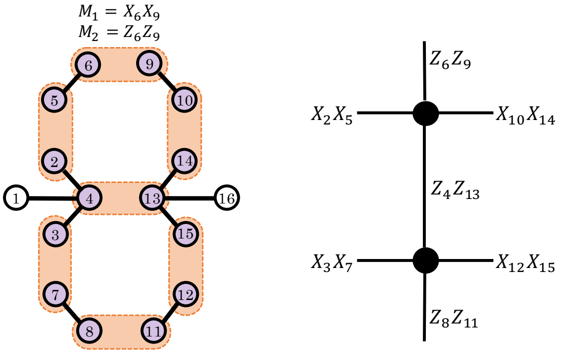

(a) Fusion network

(b) Syndrome graph

Resource States, R	$\left\langle \begin{pmatrix} X_1Z_4, \\ X_2Z_4, \\ X_3Z_4, \\ X_4Z_1Z_2Z_3, \end{pmatrix}, \begin{pmatrix} X_{14}Z_{13}, \\ X_{15}Z_{13}, \\ X_{16}Z_{13}, \\ X_{13}Z_{14}Z_{15}Z_{16} \end{pmatrix}, \begin{pmatrix} X_5Z_6, \\ X_6Z_5 \end{pmatrix}, \begin{pmatrix} X_7Z_8, \\ X_8Z_7 \end{pmatrix}, \begin{pmatrix} X_9Z_{10}, \\ X_{10}Z_9 \end{pmatrix}, \begin{pmatrix} X_{11}Z_{12}, \\ X_{12}Z_{11} \end{pmatrix} \right\rangle$
Fusion, F	$\left\langle \begin{pmatrix} X_2X_5, \\ Z_2Z_5 \end{pmatrix}, \begin{pmatrix} X_3X_7, \\ Z_3Z_7 \end{pmatrix}, \begin{pmatrix} X_6X_9, \\ Z_6Z_9 \end{pmatrix}, \begin{pmatrix} X_8X_{11}, \\ Z_8Z_{11} \end{pmatrix}, \begin{pmatrix} X_4X_{13}, \\ Z_4Z_{13} \end{pmatrix}, \begin{pmatrix} X_{10}X_{14}, \\ Z_{10}Z_{14}, \end{pmatrix}, \begin{pmatrix} X_{12}X_{15}, \\ Z_{12}Z_{15}, \end{pmatrix}, -\mathbf{1} \right\rangle$
Checks, C	$\langle X_2X_5Z_6Z_9X_{10}X_{14}Z_{13}Z_4,\ X_3X_7Z_8Z_{11}X_{12}X_{15}Z_{13}Z_4 \rangle$
Surviving Stabilizer, S	$\langle C, X_1(Z_4Z_{13})X_{16},$ $Z_1(Z_2Z_5)(X_6X_9)(Z_{10}Z_{14})(Z_{12}Z_{15})(X_8X_{11})(Z_3Z_7)(X_4X_{13})Z_{16} \rangle$

(c) Stabilizer group in the fusion network

Supplementary Fig. 4: Example fault-tolerant fusion network. (a) Arrangement of resource states, represented as graph states. Qubits that will be measured during fusion are shaded, and the output qubits that remain after measurement are unshaded. Fusion measurements are shown by orange ovals. All fusions are of the type $\langle X_iX_j, Z_iZ_j \rangle$. (b) The syndrome graph corresponding to (a) with measurements corresponding to every edge labelled. Multiplying the measurement operators adjacent to the vertices gives us the generators of the check group, C. (c) Explicit definition of the generators of each stabilizer group, where the brackets in the expressions are a guide to indicate the separable sub-groups of F and R. R is the union of the stabilizers of different resource states that can be inferred from their graph state representation. The fusion group is generated by all the fusion measurements and $-\mathbf{1}$. Generators from different resources states in R and different fusions in F are sorted by column. The surviving stabilizer group, S, includes check operators, and stabilizers that include the output qubits (1,16).

Undetectable errors The group of undetectable errors is defined by the centralizer of the check group C on the whole Pauli group:

$$U := \mathcal{Z}(C). \tag{15}$$

As the name suggests, this is the subgroup of the Pauli operators which leaves no trace on the check operator results. However, not all undetectable errors are problematic for computation, since some do not affect the final correlations of interest. For example, U contains elements of R (or F) which will have no detrimental effect as they leave the resource states (or fusions) invariant. More generally, the group of trivial undetectable errors is

$$T := \mathcal{Z}(S), \tag{16}$$

where S is the surviving stabilizer group defined in supplementary note III. T includes $\langle R, F\rangle$ by definition. Undetectable errors can thus be classified by the elements of the quotient U/T. Trivial errors, that are elements of T, have no effect on the check operators, or the output stabilizers. Errors may also be non-trivial but undetectable. These errors do not affect the check operators, but do affect the output stabilizers. For fault-tolerance, we are interested in fusion networks where the weight of non-trivial undetectable errors, also called the distance of the code, increases with the size of the network. Some examples of such networks are discussed in supplementary note V.

Error representation. Errors may occur in the generation of resource states, or in the fusion measurements themselves. It is convenient to choose a representation that groups together errors which have an equivalent action on the code state and check operators. It is never necessary to distinguish different errors which are equivalent up to an element of F, since $F \subseteq T$. We therefore choose to express decoding problems in terms of elements of P/F, where P is the Pauli group (i.e. the full Pauli group quotiented by the fusion group). While distinct elements of P/F may correspond to equivalent errors (according to the fully reduced equivalence classes of P/T), the partial reduction P/F, has the advantage of preserving a large amount of the locality structure in the error model. In particular, single qubit Pauli errors on resource states are interpreted as measurement errors on a corresponding generator(s) of F. When F is composed of Bell fusion measurements, the quotient P/F identifies pairs of single qubit Paulis in P whenever they jointly produce an element of F. We can thus choose to express decoding problems in terms of P/F, which directly corresponds to specifying which fusion outcomes were flipped. For instance, in the example of Supplementary Fig. 4, the single qubit errors X_4 and X_{13} are equivalent errors, as they multiply to X_4X_{13}, which is an element of F. Up to this equivalence, errors can be characterized by which generators of F (fusion outcomes) they flip, in this case Z_4Z_{13}.

B. Example fault-tolerant fusion network

Supplementary Fig. 4 shows an example of a fusion network, where the output is a Bell pair on qubits 1 and 16. The stabilizer groups R, F, S and C for this example are defined in the table in Supplementary Fig. 4(c). This network contains two check operators, which allow certain errors in the fusion network to be corrected.

After all fusions have been performed the quantum correlations of the unmeasured qubits depend on the classical measurement information, and this dependency is captured by the surviving stabilizer group as we saw in supplementary note III. We use the notation $m^{XX}_{i,j}$ to represent the classical bit from the measurement of the operator X_iX_j and $m^{ZZ}_{i,j}$ for the measurement of Z_iZ_j.

The outcomes (eigenvalues) of the check operator generators are:

$$\begin{aligned} C = \langle & m^{XX}_{2,5} m^{ZZ}_{6,9} m^{XX}_{10,14} m^{ZZ}_{4,13}, \\ & m^{XX}_{3,7} m^{ZZ}_{8,11} m^{XX}_{12,15} m^{ZZ}_{4,13}\rangle. \end{aligned} \tag{17}$$

Since check operators should have positive eigenvalue, they act as parity checks on the bits describing the fusion outcomes. The output stabilizer group is:

$$\begin{aligned} S_{\text{out}} = \langle & m^{ZZ}_{4,13} X_1 X_{16}, \\ & m^{XX}_{4,13} m^{ZZ}_{2,5} m^{XX}_{6,9} m^{ZZ}_{10,14} m^{XX}_{12,15} m^{XX}_{8,11} m^{XX}_{3,7} Z_1 Z_{16}\rangle \end{aligned} \tag{18}$$

such that when all fusions succeed the fusion network produces a Bell pair $\langle \pm X_1X_{16}, \pm Z_1Z_{16}\rangle$.

In this small example some (but not all) fusion measurement errors can be corrected. For example, consider a measurement error in Z_4Z_{13}. The corresponding outcome, $m^{ZZ}_{4,13}$ appears in the output stabilizer, determining the sign of X_1X_{16}, which is the XX stabilizer of the output Bell state, so an error will result in an incorrect sign. However, the measurement also appears in both checks in C, and so it can be corrected.

In this example we can also identify the other types of error configuration. Trivial errors correspond to elements of T, for example an error $E_1 = Z_2Z_{10}$ will flip measurement outcomes X_2X_5 and $X_{10}X_{14}$ but neither check is affected. Since $E_1 \in R \cup F$ this has a trivial effect on the output stabilizer.

Consider another error: $E_2 = Z_4$. This is a non-trivial undetectable error, which causes a single flipped measurement in the fusion outcome X_4X_{13}. This error commutes with the check group C, which we can see by noting that $m^{XX}_{4,13}$ is not present in either of the check operators, and it will therefore go undetected. E_2 also anti-commutes with one of the additional generators in S, since we can see its presence in the output stabilizer. For this reason, this error will lead to an incorrect prediction for the sign on $\pm Z_1Z_{16}$ on the output stabilizers.

C. Topological fault-tolerant fusion networks

We can introduce a notion of low-density parity check (LDPC) fusion networks that is analogous to other similar constructions in error correction [27, 28]. Namely, a family of stabilizer FTFNs is LDPC if:

1. it has check operator generators such that each generator involves a bounded number of fusions, and each fusion is involved on a bounded number of generators,
2. for any integer d, there exist fusion networks in the family such that non-trivial undetectable errors have support on at least d qubits.

One can then apply the usual combinatorial arguments to show the existence of error thresholds [29–31]. In the FBQC setting the fusion measurement outcomes will form a classical LDPC code.

We are specifically interested here in topological LDPC fusion networks, where the operations are geometrically local, and the resulting parity checks and logical operators have the structure of fault-tolerant topological error correction. The fusion network captures the behavior of a code evolving over time, so that for 2D topological codes one obtains 3D topological fusion networks. The elements of the surviving stabilizer group S take the form of membranes (word-lines of string operators) and undetectable errors in U take the form of closed strings (word-lines of topological charges). An illustration of such a 3D fusion network is shown in Supplementary Fig. 5. The check operators are closed membranes, while the logical operators are open membranes that span the system and are supported on the output qubits. The introduction of appropriate boundary conditions to the fusion network allows these membranes to terminate, and we discuss this further in supplementary note VI. While there exists the noted equivalence between these 3D topological fusion networks and 2D topological codes, the 3D topological fusion networks are not constrained to represent foliated codes [32], in which there is a notion of a fixed code structure that persists throughout the computation. FBQC can support the more general framework of fault tolerance beyond foliation described in [33, 34]. Examples of topological fusion networks are given in supplementary note V.

D. Syndrome graphs

The codes associated to fault-tolerant fusion networks are often well described by a *syndrome graph* representation. The syndrome graph picture makes it easier to interpret the underlying structure of the code and enables the application of existing decoders such as minimum-weight matching and union-find decoders [35, 36]. However, it should be noted that not all schemes can be naturally represented by a syndrome graph structure. That this is possible in topological (surface-code based) FBQC, is a manifestation of the fact that error chains leave non-trivial syndromes only at their endpoints.

In the syndrome graph we represent the code through a multi-graph, with vertices corresponding to check operator generators in C, and each edge corresponding to a generator of the fusion group F. An edge is connected to a vertex if the corresponding generator of F is a factor of the corresponding check operator in C.

The parity for a check operator is evaluated by taking the joint parity of all the measurement outcomes composing the check. Given a set of fusion measurement outcomes each parity check has an associated parity value of either +1 or -1. The configuration of all of these parity outcomes is called the *syndrome*. If a fusion outcome is flipped, the vertices (checks) connected by its edge in the graph will have their parity values flipped. If a fusion outcome is erased, the two checks connected by the edge in the graph can be combined into a single check operator. The syndrome graph may have 'dangling edges', where an edge connects to only one check vertex. In some cases there may also be multi-edges between two check nodes when multiple fusion measurement outcomes contribute to the same pair of syndromes.

Supplementary Fig. 4(b) shows how the fusion network example can be represented as a syndrome graph. In this simple example there are two check operators, each check operator has four incident edges. There is one edge that is shared by both check operators, connecting the two vertices. The other edges are 'dangling edges' connected only to a single check node. In this small example not all fusion outcomes are included in a check operator, but in the examples of topological fault-tolerant fusion networks we present in the next supplementary note, all fusion outcomes will be part of at least one check operator. Specifically, we will consider topological fusion networks based on the surface code. Like any surface code construction, these networks can be represented by a *primal syndrome graph* and a *dual syndrome graph* which are locally disconnected. In the bulk, every Bell fusion contributes two measurement outcomes, which respectively correspond to one edge of the primal and one of the dual syndrome graph.

Supplementary Note V: Example Fault-Tolerant Fusion Networks

In this supplementary note, we describe two explicit examples of fault-tolerant fusion networks that implement surface code error correction. The first model is closely related to existing schemes in the literature for constructing cluster states for fault

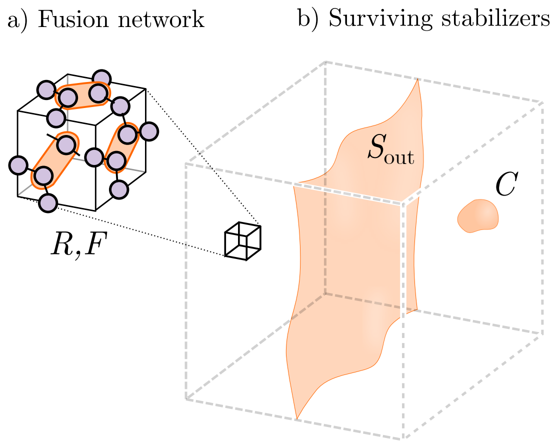

Supplementary Fig. 5: Illustration of the groups in a 3D topological fault-tolerant fusion network. Resource state group, R; Fusion group, F; Check group, C; Output statbilizer, S_{out} (a) Microscopically the fusion network is composed of resource states and fusions, which may form repeating unit cells. (b) After the measurements of the network are made we are left with a 3D block of classical information, with remaining qubits on the surface of the block. The check operators form closed surfaces in the bulk. The remaining generators of the surviving stabilizer group represent extensive membranes that span the network, these behave as logical operators.

tolerant MBQC [37, 38] while the second example shows a novel scheme which follows design principles to minimize the vertex degree of the syndrome graph. These examples provide simple illustrations of how fault-tolerance can be achieved in the FBQC framework. They are chosen as helpful pedagogical examples and are not optimal FBQC architectures. However, even with these examples we demonstrate a significant performance improvement.

A. 4-star fusion network

The '4-star' fusion network is shown in Supplementary Fig. 6. The resource state is the four qubit Greenberger-Horne-Zeilinger (GHZ) state[5] with stabilizer generators $\langle Z_1Z_2Z_3Z_4, X_1X_2, X_2X_3, X_3X_4\rangle$. For graphical clarity, we represent this resource state as a 5-qubit star graph state with the central qubit blacked out (Supplementary Fig. 6(a)) because this is the state obtained on measuring the central qubit of a 5-star graph state in the X basis with a '+1' outcome. There is no need to prepare a 5 qubit physical resource state, the 4-GHZ state can be created directly[6]. The four shaded circles, correspond to qubits in the resource state and are input to fusions in the network. The fusion network can be built up from a cubic unit cell as shown in Supplementary Fig. 6(a) and (d), where a resource state is placed on every face and edge of the unit cell. Resource states are aligned parallel to faces or perpendicular to edges. A fusion measurement is made on pairs of qubits from resource states centered at unit cell faces and qubits from resource states centered at neighboring edges as shown in Supplementary Fig. 6(b)[7]. Each fusion attempts to measure the stabilizer operators X_1Z_2 and Z_1X_2 as shown in Supplementary Fig. 6(c). We include a formal definition of the layout later in this subsection.

For each unit cell there is a primal check operator that is associated with the cell, which is made up of 24 fusion measurement outcomes. There are 4 fusions per face of this cell, giving a total of 24 by combining all 6 faces of the cube. For each of these fusions, one of the two measurement outcomes contributes to the cell parity check. Specifically the parity check operator is $C_c = \prod_{f\in c}\prod_{e\in f} Z_f X_e$, where c is the cell, f is a face, and e is an edge. The other measurement outcomes contribute to one of the dual parity checks, which are associated with the corner vertices of the unit cell. The fusion network is symmetric under translation by half the lattice constant in all three dimensions. This means that the primal syndrome graph and dual syndrome graph are identical.

The logical operators are defined by 2D membranes made up of connected faces of the lattice. To evaluate the membrane we combine the fusion measurement outcomes from all four fusions on each face that makes up the membrane. Specifically the operator for a membrane, $\mathcal{M}$, is defined as $\prod_{f\in\mathcal{M}}\prod_{e\in f} Z_f X_e$.

---

[5] For convenience in defining the fusion network our resource state here has the X- and Z- operators swapped compared to the usual GHZ state definition

[6] Using linear optics a 4-GHZ state can be prepared from single photons, using a variety of methods, such as those described in [13].

[7] If the center qubit in the resource state had not been measured, the fusions would result in the cluster state used in the MBQC implementation of the surface code [37, 38].

4-Star Fusion Network

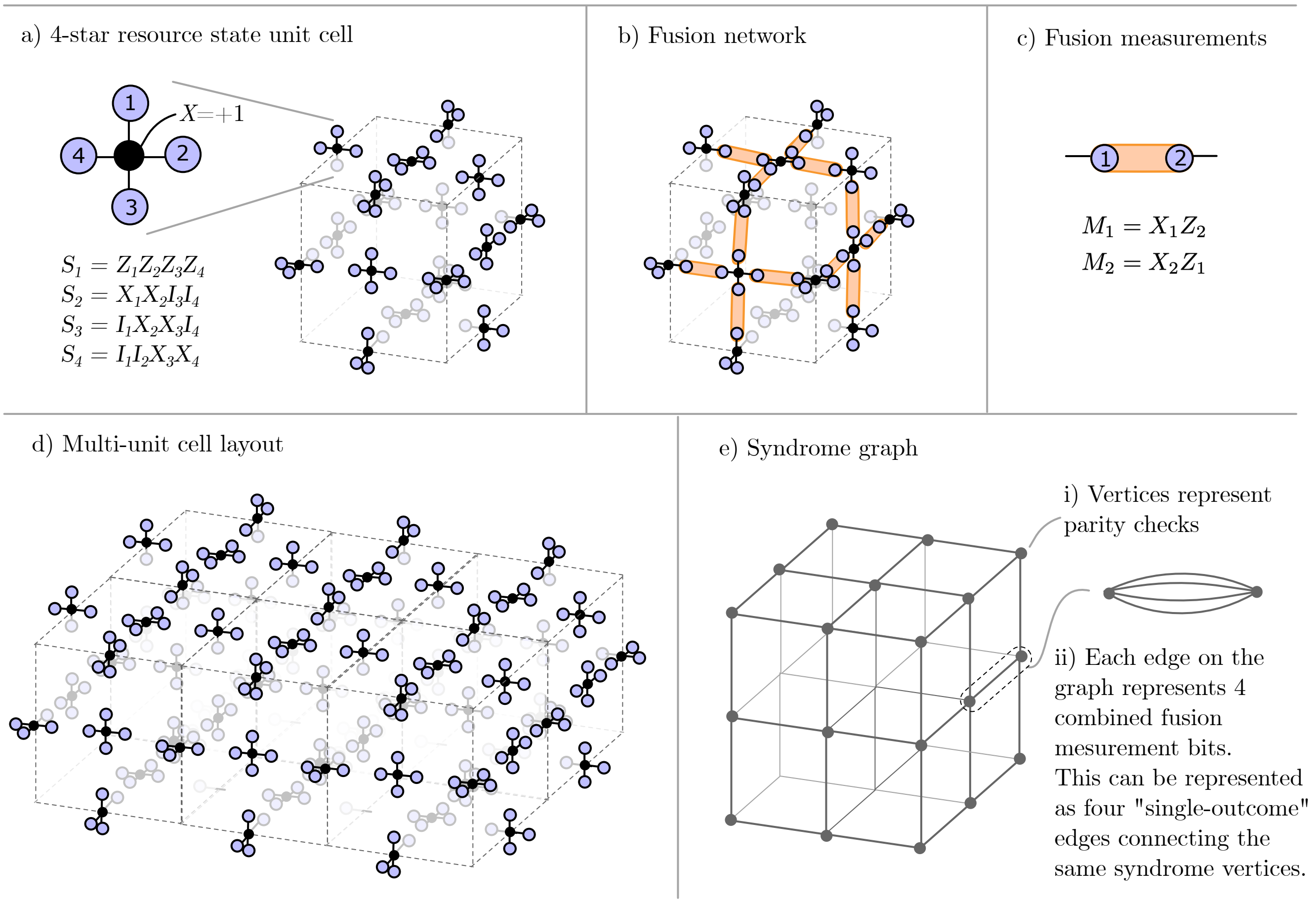

Supplementary Fig. 6: The '4-star' fusion network: (a) Layout of resource states in a cubic unit cell of the lattice (grey dashed line). The resource state is a 4-GHZ state with stabilizers $Z_1Z_2Z_3Z_4$, X_1X_2, X_2X_3, X_3X_4, which is the same as a graph state in the form of a 4-star where the central qubit (shown in black) has been measured in the X basis and returned a '+1' outcome. The resource state is drawn in this way only for ease of depiction; the architecture only requires 4-GHZ resource states which, in the case of a linear optical implementation, can be created directly from single photons using circuits described in [13] . (b) Fusions within a unit cell shown in orange (c) All fusion measurements in the fusion network attempt to measure $M_1 = X_1Z_2$ and $M_2 = Z_1X_2$. (d) Layout of resource states across multiple unit cells. (e) Syndrome graph resulting from the fusion layout. Both the primal and dual syndrome graphs have the same structure. Primal and dual checks can respectively be associated to unit cubes and vertices of the dashed lattice of (d). Individual checks can be obtained by taking the product of 24 fusions between adjacent edge-face pairs (whether XZ or ZX is used for respectively for primal or dual).

The fusion network results in the syndrome graph shown in Supplementary Fig. 6(e): a cubic lattice, where every edge is a multi-edge corresponding to four measurement outcomes. In total there are 24 fusion measurements that combine to evaluate each check operator. Since the syndrome graph is used across different implementations of the surface code, it is a useful tool to understand the correspondence between FBQC and a circuit based surface code model. In a circuit model, space-like edges in the 3D syndrome graph correspond to physical qubit errors, while time-like edges correspond to measurement errors. In FBQC both time-like and space-like edges correspond to fusion measurement outcomes - there is no distinction between physical and measurement errors in this model. Another point of comparison is the interpretation of the primal and dual syndrome graphs. In a circuit model the primal syndrome graph captures Pauli-X errors, and measurement errors on Z-type parity checks, while the dual syndrome graph captures Pauli-Z errors and measurement errors on X-type checks. In this FBQC example, each 2-qubit fusion contributes one measurement outcome to the primal graph, and the other to the dual graph. One way of viewing this is that the two fusion measurement outcomes behave like the Pauli-X and Pauli-Z parts of the error channel on a physical qubit in the circuit model.

For a fusion network like this, that generates the bulk of a fault tolerance structure, it is always possible to swap the resource states and measurements to find another valid fault tolerant fusion network, i.e. defining $R' = M$ and $M' = R$. In this case that would mean that the resource states become bell pairs, and the fusion measurements become 4-qubit projective measurements on a GHZ state. Depending on the hardware available, one or the other of these fusion networks may be preferable.

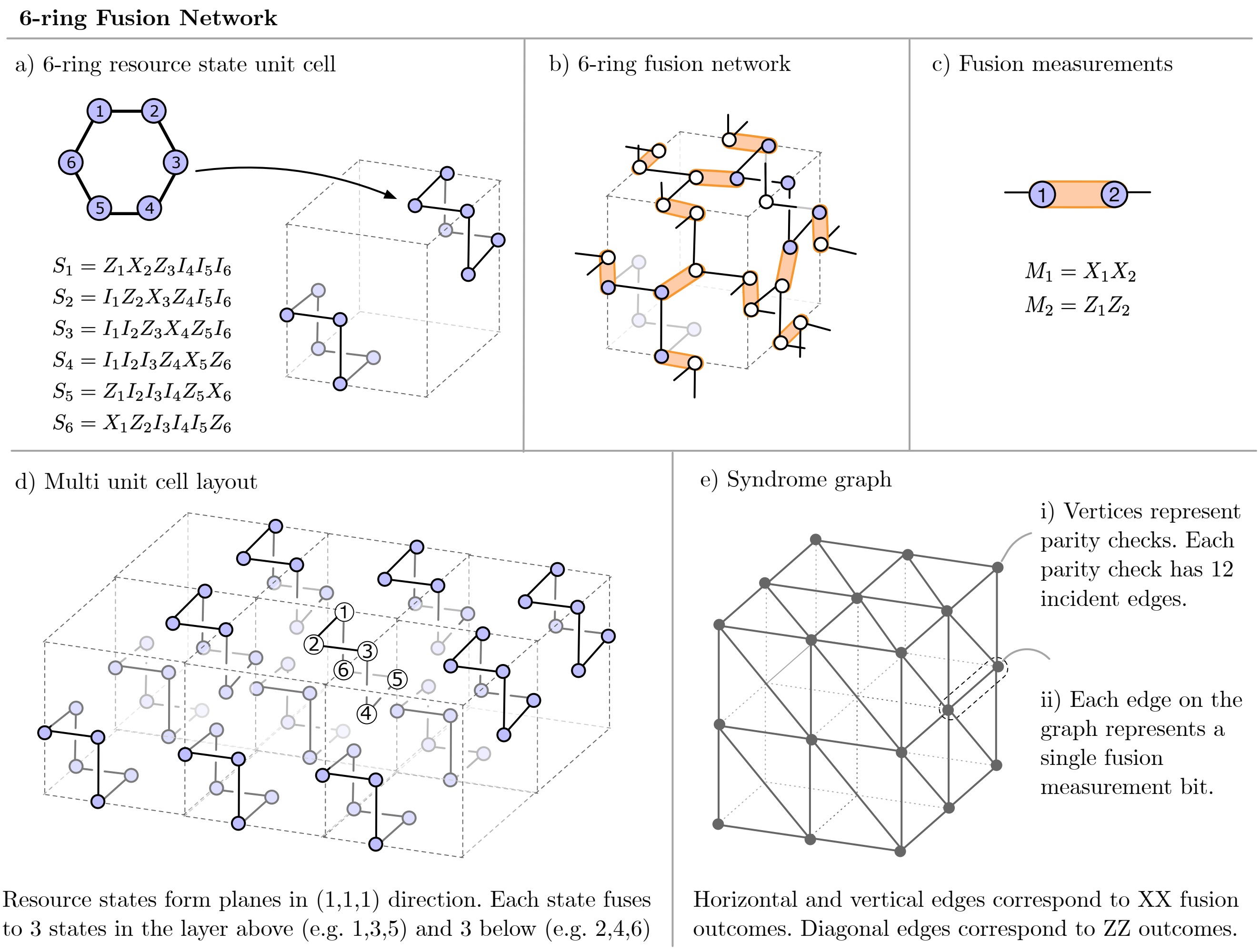

Supplementary Fig. 7: The '6-ring' fusion network. (a) Each resource state is a graph state in the form of a ring of six qubits. Two resource states are placed at opposite corners of each unit cell. (b) Two-qubit fusions connect every pair of qubits that share a face or an edge. Resource states that belong to the unit cell are shown as shaded circles, while qubits from resource states in neighboring cells are shown as white circles. A formal definition of the fusion network can be found in the text. (c) All fusion measurements in the fusion network are two-qubit projective measurements on the bases $M_1 = X_1X_2$ and $M_2 = Z_1Z_2$. (d) Shows the layout of resource states across multiple unit cells. When unit cells are tiled the resource states can be grouped into layers along 2D planes perpendicular to the (1,1,1) direction. Three qubits in each state fuse with the layer above, and three with the layer below. (e) The syndrome graph resulting from the fusion layout is a cubic graph with diagonal edges as shown. Primal and dual syndrome graphs have an identical structure. In both, the vertical edges correspond to XX type fusion outcomes and diagonal edges correspond to ZZ outcomes.

We now give an explicit geometric definition of the 4-star fusion network to accompany Supplementary Fig. 6. The geometry is merely a tool for defining the connectivity of the fusion network, and the physical positioning on the qubits and fusions has no intrinsic meaning. Each cubic unit cell contains 6 resource states. Three are positioned on unit cell edges at locations (1/2, 0,0), (0,1/2,0) and (0,0,1/2). Three are positioned on unit cell faces at locations (1/2,1/2,0), (1/2,0,1/2) and (0,1/2,1/2). Each resource state has four qubits, which are all equivalent. In addition the unit cell contains 12 two-qubit fusion operations. Every resource state positioned at a face undergoes a fusion with the resource state located at the four edges making up the boundary of the face. All fusions are a stabilizer measurement of the operators XZ and ZX on the two measured qubits.

B. 6-ring fusion network

Our second example, the 6-ring fusion network, improves on the 4-star network. It requires fewer resource states and fewer fusion measurements to implement a code of the same distance, and as shown in the main paper, it offers a significantly improved threshold.

In this fusion network, the resource states are graph states in the form of six qubit rings, with stabilizers $\langle Z_1X_2Z_3, Z_2X_3Z_4, Z_3X_4Z_5, Z_4X_5Z_6, Z_5X_6Z_1, Z_6X_1Z_2\rangle$. The fusion network has a cubic unit cell with two resource states per unit cell, as depicted in Supplementary Fig. 7(a). Fusion measurements connect the pair of qubits at each face and each edge, as shown by the orange lines in Supplementary Fig. 7(b). Each fusion attempts to measure the stabilizer operators X_1X_2 and Z_1Z_2 on the input qubits. Supplementary Fig. 7(d) shows multiple unit cells, where it can be seen that the resource states form layers in the planes perpendicular to the (1,1,1) direction. We present a formal definition of the 6-ring fusion network later in this subsection.

There are 12 fusion measurements that combine to evaluate each check, which is half as many measurements per check compared to the 4-star network. For each unit cell there is a primal check operator that is associated with the cell. For each cell, the check operator comprises measurements for one fusion per face, and additionally fusions for 6 edges of the cell. The edges that contribute to the cells check are the 6 edges that do not have a qubit from a resource state that belongs to the cell, as represented in Supplementary Fig. 7(a). At these edges a fusion measurement is needed to link together the stabilizers of neighboring faces, we call this set of edges $E_{\mathrm{link,c}}$. For each of these fusions, one of the two measurement outcomes contributes to the cell parity check. Specifically the parity check operator is $C_c = \prod_{f\in c}(XX)_f \prod_{e\in E_{\mathrm{link,c}}}(ZZ)_e$, where c is the cell, f is a face, and e is an edge. The other measurement outcomes contribute to one of the dual parity checks, which are associated with the corner vertices of the unit cell.

The logical operators are defined by 2D membranes made up of connected faces of the lattice. To evaluate the membrane we combine the fusion measurement outcomes from the fusion on each face, and the fusion at each connection between two faces. For example if we consider a flat 2D sheet membrane, $\mathcal{M}$, then we can define the membrane operator as $\prod_{f\in\mathcal{M}}(XX)_f \prod_{e\in\mathcal{M}}(ZZ)_e$. Other representations of the logical membrane can be obtained by multiplying by check operators. In some configurations two connected faces of the membrane may be connected through a resource state, in which can the fusion at this edge is not included in the membrane operator.

The syndrome graph for this fusion network is depicted in Supplementary Fig. 7(e), and is a cubic lattice with added diagonal edges. The 6-ring network has the same symmetry under translation in all three dimensions by half the lattice constant. So, as in the 4-star network, the primal and dual syndrome graphs are identical.

The diagonal edges that show up in the syndrome graph here are a familiar feature in circuit based surface codes, where they would be interpreted as so-called 'hook' errors. These can occur when a single error event spreads to neighboring qubits during the stabilizer measurement circuit. Although the origin of this type of correlated error is very different in the fusion network setting, in both cases these edges arise due to the process of creating large scale entanglement from physically low weight operations.

We now give an explicit geometric definition of the 6-ring fusion network to accompany Supplementary Fig. 7. Resource states are distributed according to a body-centered cubic lattice, centered at locations (0,0,0), (1/2,1/2,1/2) in the unit cell. Additionally, we associate individual coordinates to the qubits of the resource states according to the mapping, where these positions are relative to the resource state position:

$$\begin{aligned}
&1 : (1/4, 0, 0),\\
&2 : (1/4, 1/4, 0),\\
&3 : (0, 1/4, 0),\\
&4 : (0, 1/4, 1/4),\\
&5 : (0, 0, 1/4),\\
&6 : (1/4, 0, 1/4).
\end{aligned}$$

There are two qubits located at each edge, which are connected by a fusion, and two qubits located at each face, which are also connected by a fusion.

C. Rotated 6-ring fusion network

In supplementary note V B above we introduced the 6-ring fusion network in a form that shows its relationship to the surface code, where it is defined on a cubic lattice which is the cell complex that underlies the structure of the topological fault tolerance of the system. For considering the physical architecture and performing simulations we make use of a different representation which rotates the structure in space-time. Locally this is identical to the fusion network described above and in the main text, but differs in the definition of non-local periodic boundary conditions. In the rotated version we consider another cubic graph as shown in Supplementary Fig. 8, where now every cubic cell corresponds to one resource state. Each resource state is fused with the resource states corresponding to the 6 other cells with which it shares a face, and therefore each face corresponds to a fusion. In this configuration every vertex of the graph corresponds to a parity check, and the vertices are two colorable, with the vertices

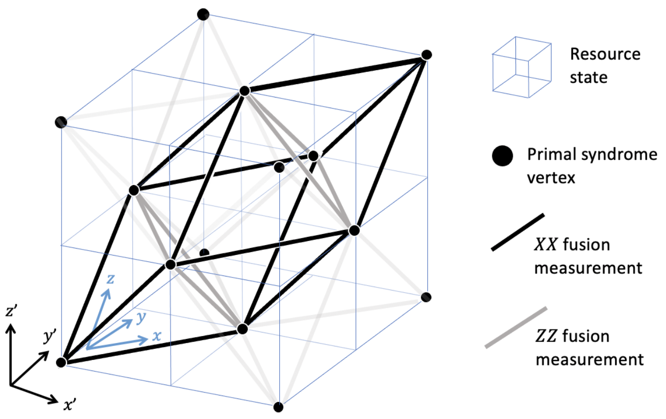

Supplementary Fig. 8: Rotated version of the 6-ring fusion network, and its syndrome graph.

of a each color corresponding to either the primal or dual parity checks. In Supplementary Fig. 8 the vertices corresponding to primal checks are highlighted with black circles. The edges of the syndrome graph are diagonal edges across each face, and there are two such edges for each face, one of which will contribute to the primal syndrome graph and the other to the dual. In the Figure we show one unit cell of the cell-complex representation used in the main text highlighted in the rotated format. The x, y, z directions are transformed such that $x = x' + y'$, $y = x' + z'$ and $z = z' + y'$. In this configuration it is clear that the resource states can be arranged in a cubic configuration and must fuse with their 6 neighbors. This is the feature we exploit to construct the physical architecture shown in Figure 5 in the main paper, where time is used to simulate the z' dimension. We use the same configuration when we simulate the performance of the fusion network, and we define a 3d block of the fusion network with periodic boundary conditions in the x', y' and z' directions.

Supplementary Note VI: Quantum computation with fault-tolerant fusion networks

The previous supplementary notes have described how to create a fault tolerant *bulk* in FBQC - which behaves as the fabric of topological quantum computation. Creating the bulk is the most critical component of the architecture, as it is this that determines the error correction threshold. But to implement fault-tolerant *computation*, additional features are needed. We now turn to the question of how this bulk can be used to implement fault-tolerant logic, and the implications for classical processing and physical architecture. Here we provide a brief overview of logic in FBQC and architectural considerations, both of which are topics in their own right and which are explored in much greater depth in related publications [21, 39].

A. Logical Gates

In order to perform fault-tolerant logic, we need to add to our toolkit the ability to create *topological features* in addition to the bulk. There are different approaches that can be used to create a fault-tolerant Clifford gate set. Boundaries can be used to create punctures which can be braided to perform gates via code deformation [40, 41]. Boundaries can be used to create patches on which lattice surgery can be performed [42]. Logical qubits can alternatively be encoded in defects and twists [26, 43, 44]. All of these approaches to logic are compatible with FBQC. The necessary topological features can be created by modifying fusion measurements in certain locations, or adding single qubit measurements in an appropriate configuration. Here we give a simple example of how to create two types of boundaries, which is sufficient to enable the encoding and manipulation of logical qubits in punctures or patches. Other topological features are addressed in [39].

1. Boundary creation

These two boundary types we create correspond to rough and smooth boundaries in the surface code picture [45], but in FBQC it is more natural to refer to them as primal and dual boundaries, according to whether they are able to match excitations in the primal/dual syndrome graphs respectively. A primal boundary corresponds to a rough boundary in the primal syndrome graph, and to a smooth boundary in the dual syndrome graph.

Supplementary Fig. 9 illustrates a simple example of how primal and dual boundaries can be created by measuring certain

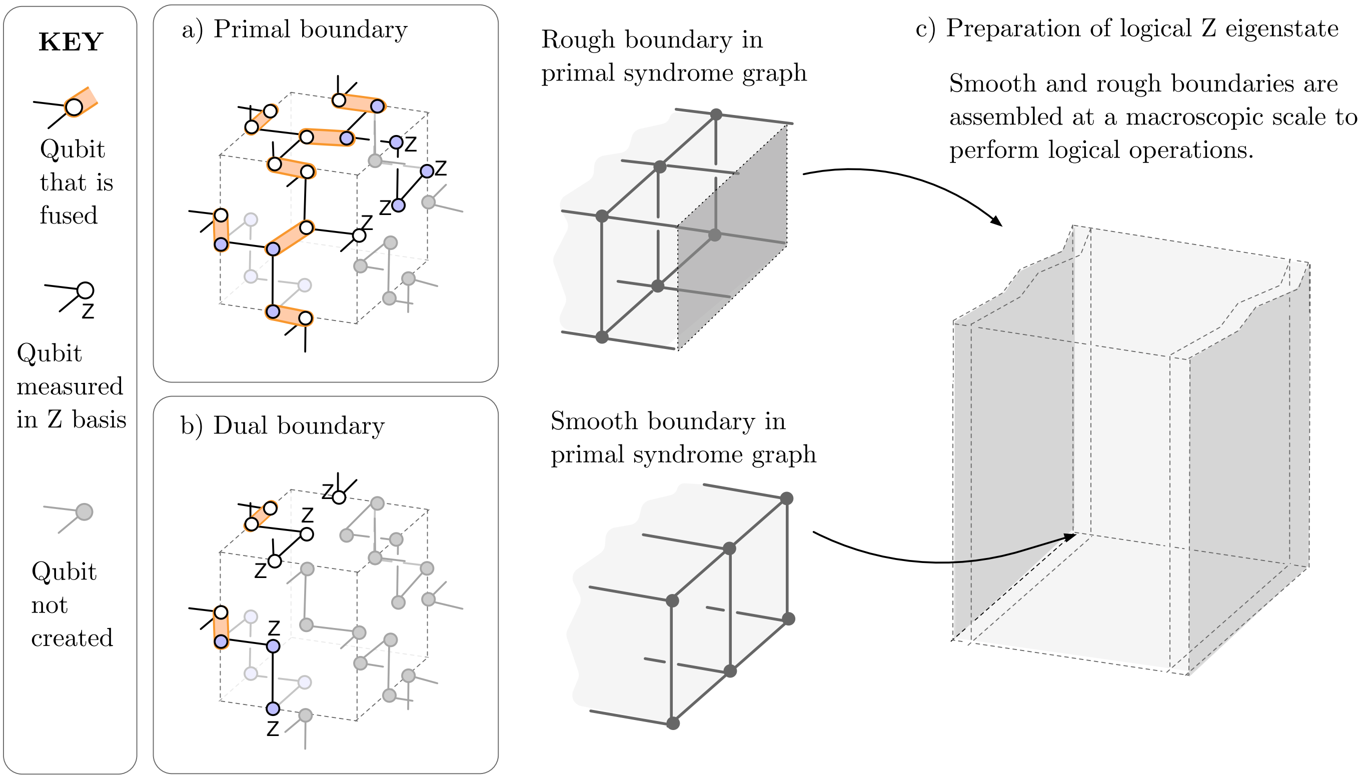

Supplementary Fig. 9: A scheme for creating primal and dual boundaries that can be used to modify the bulk to perform quantum computation. a) and b) show modified unit cells of a fusion network that can generate a primal and dual boundary respectively. In each, the fusion network is made up of the same configuration of resource states as in the bulk (see Supplementary Fig. 7), but where some subset of the fusion measurements have been replaced with single qubit Z measurements, and some subset of the resource states are entirely removed (indicated by greyed out circles). If at the boundary a resource state has no remaining entangling operations connecting it into the bulk, then it does not need to be created. All the remaining fusions (shown by orange ovals) are a projective measurement on XX, and ZZ. The effect of this modified network is to truncate the bulk either at (a) a slice halfway through the cell or (b) at the edge of the cell. A primal (dual) boundary terminates a primal (dual) logical membrane, and therefore has a rough boundary in the primal (dual) syndrome graph. The primal (dual) boundary has a smooth boundary in the dual (primal) syndrome graph. (c) Shows an example of hows these unit cells can be composed to create macroscopic boundary conditions, enabling fault-tolerant logic. This shows logical block that corresponds to fault-tolerant preparation of a dual membrane.

qubits of resource states in the Z basis. Supplementary Fig. 9(a) shows the creation of a boundary, where the layer of qubits at the boundary of the unit cell are either measured in the Z basis, or simply never created. Supplementary Fig. 9(b) shows a similar protocol for creating a smooth boundary. The case in which boundaries are parallel to planes defined by pairs of the unit cell vectors are particularly simple. For such boundaries, the only distinction between the primal and dual case is a displacement by half a unit vector in the perpendicular direction. The effect of this measurement pattern is to terminate the bulk, creating boundaries which can then be used as a feature to encode and manipulate logical qubits. Supplementary Fig. 9(c) shows an example of how these boundaries can be macroscopically assembled to fault tolerantly prepare the state $|0\rangle$ (or $|1\rangle$) in a patch encoded logical qubit.

2. *Pauli frame tracking*

In FBQC, logical states have a direct physical counterpart only up to a Pauli correction, which is tracked in classical logic through the so called *Pauli frame*. The use of a Pauli frame is necessary due to the intrinsic randomness which is introduced by teleportations carried out by Bell measurements. For instance, at the logical level, the same component which prepares a $|0\rangle$ state in Supplementary Fig. 9 will also represent the preparation of a $|1\rangle \equiv X|0\rangle$ state. In general, this means [46, 47] that a state $|\psi\rangle$ may be physically represented by a different state $P|\psi\rangle$ for some tracked Pauli correction operator P.

When relying on Pauli frame tracking, a generic n qubit state can be represented by any of 4^n possible physical quantum states together with $2n$ classical bits which describe frame. The use of stabilizer codes which protect the logical information practically halves the number of bits required to describe the frame. The key property of this technique is that most of the computation which can be described by Clifford operations can be executed independently of the classical tracking information.

The classical Pauli frame data only influences the quantum operations performed at the logical level, in cases such as magic state injection and distillation. This allows classical Pauli frame processing to occur at a logical clock rate rather than at a potentially much faster physical fusion clock rate. In supplementary note VIII, we explain Pauli frame tracking for fault-tolerant FBQC, explaining why this technique only imposes minimal quantum and classical processing requirements.

3. Universal logic

To achieve a universal gate set, the Clifford gates must be supplemented with state injection, which combined with magic state distillation protocols can be used to implement T gates, or other small angle rotation gates. Magic state injection can be implemented in FBQC by performing a modified fusion operation, by making a single qubit $\frac{\pi}{8}$ measurement, or by replacing a resource state with a special 'magic' resource state.

B. Decoding and other classical processing

In FBQC, as in other approaches to fault-tolerant quantum computation, classical error-correction protocols are in charge of extracting reliable logical measurement information from the unreliable and noisy physical measurement outcomes. In FBQC it is helpful to view the decoding outcomes as *logical Pauli frame information*. Keeping track of this logical Pauli frame is necessary to interpret future measurement outcomes.

This logical Pauli frame produces time-sensitive information when logical level feed-forward is required. That is, when a logical measurement outcome is used to decide on a future logical gate it is necessary to have the relevant Pauli frame information available. One example of this is for the realization of T-gates via magic state injection where a S or $S^\dagger$ is applied conditioned on a logical measurement outcome.

One widely discussed challenge of decoding is that it must be performed live during quantum computation. However, it is a crucial feature that this feed-forward operation happens at the *logical timescale*, and decoding outcomes are not needed at the fusion (or physical qubit) timescale. If decoding is slower than the logical clock rate then *buffering* or *ancillary logical qubits* [48] can be used to allow the computation to 'wait' for the decoding outcomes. We discuss this further in supplementary note IX. It is worth emphasizing that these are tools used at the logical level, and it is never necessary to modify any physical operation. Fusions can always proceed without decoding outcomes. An important implication of this is that a slow decoder does not impact threshold. Nevertheless fast decoders are desirable to reduce unnecessary overhead.

Supplementary Note VII: Simulation methods

The hardware-agnostic fusion error model is an i.i.d error model in which every measurement outcome from every fusion is independently erased with probability p_{erasure} and suffers a Pauli error (i.e. the measurement outcome is flipped) with probability p_{error}. Since a measurement can only be incorrect if it is not erased, the probability of the three measurement outcomes are as follows:

- Correct measurement: $(1-p_{\mathrm{erasure}})(1-p_{\mathrm{error}})$
- Erasure: p_{erasure}
- Incorrect measurement: $p_{\mathrm{error}}(1-p_{\mathrm{erasure}})$

To obtain the correctable regions shown in Fig. 3 in the main paper, we perform montecarlo simulations of the behavior of the fusion network for different values of p_{erasure} and p_{error}. For each combination of error parameters we simulate a 3D block of the fusion network with periodic boundary conditions of size L_{code} =12,16, 20 resource states in each dimension. To perform a single numerical trial we sample errors and erasures on the edges of the primal and dual syndrome graph according to to the probability distribution above, and compute the resultant syndrome. We then perform decoding using the minimum weight matching decoder on the syndrome graph. We use the Blossom V implementation of graph perfect matching [49]. To construct the matching graph we assign a weight 0 to erased edges in the syndrome graph, and all other edges weight 1. We construct a complete matching graph, with a vertex for each odd parity syndrome vertex, and an edge that connects each pair of vertices where the weight of the edge corresponds to the lowest weight path connecting the two vertices on the syndrome graph. Running the decoder results in a correction, which we compare against the error sample to identify whether a logical error is introduced. We decode primal and dual syndrome graphs separately, and identify the logical error state of both after decoding. We define the overall logical error to be the case that either primal or dual syndrome graph has a logical error in any one of the three periodic dimensions. We repeat this sampling and decoding process at least 15000 times for each combination of error

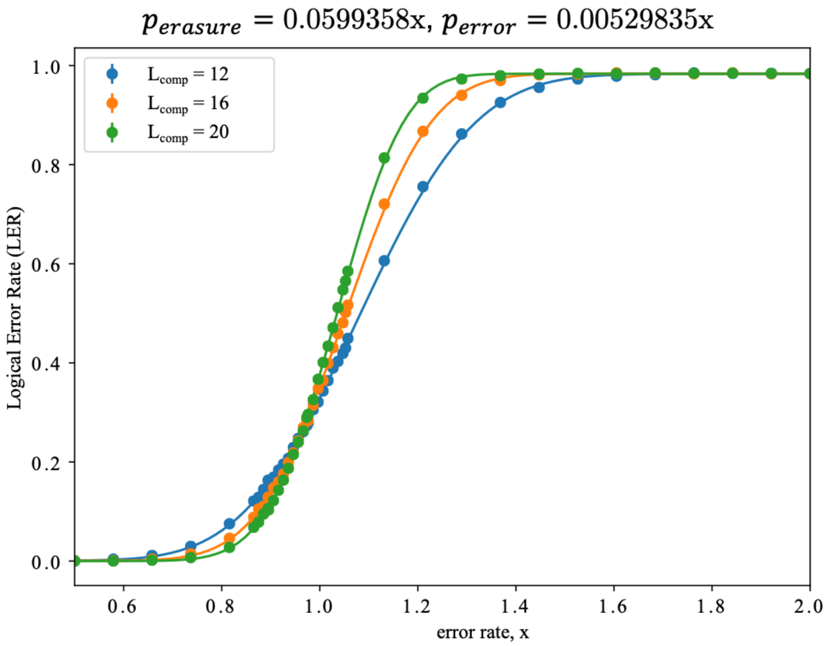

Supplementary Fig. 10: Threshold sweep under the hardware-agnostic fusion error model for the 6-ring fusion network. We set $p_{\text{erasure}} = c_{\text{erasure}}x$ and $p_{\text{error}} = c_{\text{error}}x$ with $c_{\text{erasure}} = 0.0599358$ and $c_{\text{error}} = 0.00529835$, and simulate fusion network blocks of sizes L_{code} =12,16, 20. Each marker represents one collection of trials for a single set of error parameters. The data is fit to the CDF of the beta distribution. The threshold is the intersection of the curves.

parameters to compute the logical error rate. To identify a threshold we fix the two error parameters relative to a single variable x: $p_{\text{erasure}} = c_{\text{erasure}}x$ and $p_{\text{error}} = c_{\text{error}}x$ where c_{erasure} are parameters used to vary the ratio of p_{erasure} and p_{error}. We then sweep the value of x and fit to the cumulative distribution function (CDF) of the rescaled and shifted beta distribution to identify a threshold crossing. Supplementary Fig. 10 shows such a sweep (points) and fit (lines) for the 6-ring fusion network with $c_{\text{erasure}} = 0.0599358$ and $c_{\text{error}} = 0.00529835$. The crossing of the different curves here allows us to estimate the threshold for a fixed ratio of p_{erasure} and p_{error} (equal to $c_{\text{erasure}}/c_{\text{error}}$), which represents one point in Fig. 3 in the main paper. Repeating this for different ratios of c_{erasure} and c_{error}, we map out the full curve in this figure.

In the linear optical error model, every photon in the resource state and every boosting photon has a loss probability of p_{loss}[8]. Every fusion has a failure probability p_{fail} which determines the level of boosting required in the fusion and the number of photons involved in each fusion. Following the randomized failure model in supplementary note II B, we find that the erasure probability of both the XX and ZZ measurements for every physical fusion is p_0 (eq. 4). If the qubits in the resource state are unencoded, this is the erasure probability of every measurement in the fusion network. If the resource state is encoded in a (2,2)-Shor code as described in supplementary note II C, the erasure probability of every measurement in the fusion network is equal to p_{enc} (eq. 5). Solving the equation $p_{\text{enc}} = p^*_{\text{erasure}}$ ($p_0 = p^*_{\text{erasure}}$) for p_{loss}, where p^*_{erasure}, is the erasure threshold of the fusion network, gives us the loss threshold of the fusion network with encoded (unencoded) resource states for a specific value of p_{fail}. Repeating this for many values of p_{fail} gives us the curves in Fig. 6 in the main paper.

Supplementary Note VIII: Pauli frame

During a fusion-based computation we must constantly keep track of the Pauli frame of each logical qubit. After every logical gate, the Pauli frame is updated.

---

[8] When fully accounting for the effect of hardware level errors on photon loss, the loss probabilities of photons used for boosting is likely to be lower than the loss on photons in the resource state. The model we have used is conservative in this respect.

a. The Pauli Frame

The *Pauli frame* representation of quantum state consists of representing each n-qubit quantum state ψ through an n-qubit Pauli operator P and a state ψ' such that $\psi = P\psi' P^{\dagger}$. The Pauli group on n qubits comprises 4^n elements, so P can be kept track of classically using $2n$ bits.

The elements in a Pauli orbit of a given stabilizer state, S, have the same stabilizer group up to signs. It is this sign that we need to know about during a computation to be able to correctly interpret measurements on logical qubits.

b. Where do non-trivial Pauli frames come from.

Pauli frames emerge during a computation in any architecture for stabilizer-based fault-tolerant quantum computing due to accumulation of physical errors. Decoding syndrome information identifies a correction. It is not necessary to physically implement this correction but rather this can be tracked in the Pauli frame.

In FBQC we must additionally account for the fact that measurement outcomes themselves are intrinsically random. Contrary to the unitary circuit picture, for which (in absence of errors), there is a unique and well defined instantaneous quantum state of the computation ψ_t at any point in time t, this is not the case for FBQC. As in MBQC, even ideal computations in FBQC proceed with non-determinism in individual measurement outcomes. In our setting this is due to the fusion measurements which are used to propagate entanglement and correlations through the computation. Ideally, each Bell fusion can project onto one of four orthogonal entangled states $\{\langle\Phi^+|, \langle\Phi^-|, \langle\Psi^+|, \langle\Psi^-|\}$ which are equivalent up to the application of a single qubit Pauli operator. A possible treatment of these fusions is to interpret them as a canonical projection (say, $\langle\Phi^+|$) and 'teleport' an outcome dependent Pauli onto the unmeasured qubits. This is possible assuming that the original state $|\psi\rangle$ being projected has a stabilizer S which can compensate the necessary Pauli on one of the measured qubits.

In fault-tolerant FBQC, the Pauli frame tracks a combination of both of these effects. a) the Pauli correction originating from the intrinsic randomness as well as b) the Pauli correction associated to the most likely Pauli fault equivalence class associated to the extracted syndrome information.

c. Logical Clifford Gates

Since logical Pauli operations simply correspond to a change in the Pauli frame, they need not be applied explicitly at the quantum level. Instead they can simply be kept track of using the classical Pauli frame register [47]. More generally, whenever we wish to implement a quantum gate U on a state $\psi = P\psi' P^{\dagger}$, we can physically implement $U' \equiv QUP^{\dagger}$ for some conveniently chosen Pauli operator Q which will be the new Pauli frame after the gate is implemented. If U is a Clifford operation, there is no additional cost to this in terms of quantum operations, as the Pauli Q can be chosen to be $Q := UPU^{\dagger}$, such that $U' := U$ can be implemented. Pauli product measurements[48] are similarly unaffected by the presence of a Pauli frame. The fact that that the same Clifford operations U can be used regardless of the specific Pauli frame is of exceptional importance. It implies that the Pauli frame tracking may lag with respect to the application of the operations themselves, as we discussed in supplementary note IX.

The Pauli frame is needed when it comes to logical readout. We must know the Pauli frame in order to correctly interpret logical measurement outcomes.

Supplementary Note IX: Decoding

The challenges of decoding and handling classical processing and feedforward at the logical level are shared between all models of quantum computation. As we discussed in the main text, FBQC allows a separation of timescales, such that this logical level classical processing can be separated from the timescales of physical measurement reconfiguration. Nevertheless, in the very probable case that decoding and other logical level computation is slower than the physical clock cycle time, the latency of this computation must be accounted for. This can be done through a combination of modifying the logical circuit to allow for decoding latency, and adding additional processors to increase throughput. To illustrate this we can consider the example of the logical feedforward that is needed for a magic state injection circuit. In this case, the state injection involves coupling a target logical qubit with a distilled magic state, making a logical measurement, and then performing a correction circuit dependent on the logical outcome of that measurement. In order to read out the logical measurement we must have completed the decoding up until that point in order to interpret the measurement outcomes. The information needs to become available before the correction circuit is implemented. Supplementary Fig. 11 illustrates this sequence of events. The crucial timescale here is the latency between receiving the last piece of physical measurement information needed to decode the state injection measurement, and that result being available to configure the circuit to implement the correction circuit. This latency includes the processing time of the decoder, but also the signal transmission times, and the algorithmic level logic about how the decoder outcome will affect the future logical circuit. If this *logical latency* is longer than the physical clock cycle then we can add a buffer region which implements the identity gate until the computation is complete. It is not necessary to have completed decoding of the identity operation before implementing the correction. If the logical latency is slower than the logical clock speed then in addition to the buffer we need to include additional decoding processors to increase the throughput such that the decoding can be performed at a rate that can 'keep up' with the information being produced. The example in Supplementary Fig. 11 shows the case that the decoding time is roughly twice the logical clock time. To increase the throughput to match the

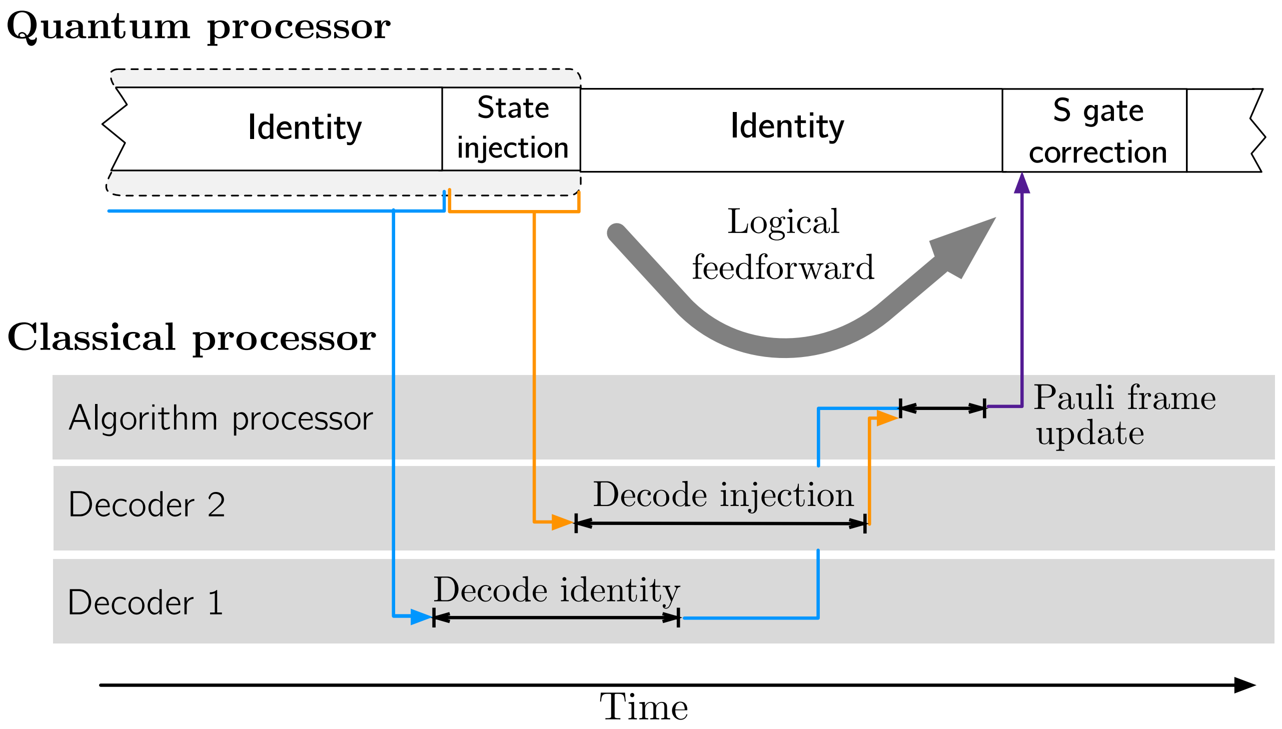

Supplementary Fig. 11: Logical feedfoward in a system where the decoding runtime is roughly twice as long as the logical clock speed. To enable logical feedforward in this situation we can increase the throughput of the decoder by including two decoder processors. In a state injection circuit a logical measurement is made. To interpret this measurement outcome we must combine the decoder outcomes from previous timesteps (indicated by the blue line) with the decoder outcome from the state injection measurement (indicated by the orange line). These solutions can be decoded and then combined to compute the overall Pauli frame, and used to control the correction circuit to complete the state injection. To allow time for this process the logical circuit includes an identity gate which essentially leaves the logical qubit to wait in memory until the information becomes available to implement the next logical gate. It is not necessary to have completed decoding of the second identity operation before implementing the S gate correction.

quantum system, we need two decoder processors per logical qubit and we need to pass data to them in an alternating fashion.

[1] Daniel Gottesman. Class of quantum error-correcting codes saturating the quantum hamming bound. *Physical Review A*, 54(3):1862, 1996.
[2] Marc Hein, Jens Eisert, and Hans J. Briegel. Multiparty entanglement in graph states. *Physical Review A*, 69(6):062311, 2004.
[3] Peter W. Shor. Scheme for reducing decoherence in quantum computer memory. *Physical review A*, 52(4):R2493, 1995.
[4] Juan I. Cirac and Peter Zoller. Quantum computations with cold trapped ions. *Physical review letters*, 74(20):4091, 1995.
[5] Klaus Mølmer and Anders Sørensen. Multiparticle entanglement of hot trapped ions. *Physical Review Letters*, 82(9):1835, 1999.
[6] Bruce E. Kane. A silicon-based nuclear spin quantum computer. *nature*, 393(6681):133–137, 1998.
[7] Daniel Loss and David P. DiVincenzo. Quantum computation with quantum dots. *Physical Review A*, 57(1):120, 1998.
[8] Jens Koch, Terri M. Yu, Jay Gambetta, A. A. Houck, D. I. Schuster, J. Majer, Alexandre Blais, M. H. Devoret, S. M. Girvin, and R. J. Schoelkopf. Charge-insensitive qubit design derived from the cooper pair box. *Phys. Rev. A*, 76:042319, Oct 2007.
[9] Alexandre Blais, Arne L. Grimsmo, S. M. Girvin, and Andreas Wallraff. Circuit quantum electrodynamics, 2020.
[10] Frank Verstraete, Michael M. Wolf, and J. Ignacio Cirac. Quantum computation, quantum state engineering, and quantum phase transitions driven by dissipation. *0803.1447*, mar 2008.
[11] Hanna Krauter, Christine A. Muschik, Kasper Jensen, Wojciech Wasilewski, Jonas M. Petersen, J. Ignacio Cirac, and Eugene S. Polzik. Entanglement Generated by Dissipation and Steady State Entanglement of Two Macroscopic Objects. *Physical Review Letters*, 107(8):080503, aug 2011.
[12] M. J. Kastoryano, F. Reiter, and A. S. Sørensen. Dissipative preparation of entanglement in optical cavities. *Phys. Rev. Lett.*, 106:090502, Feb 2011.
[13] PsiQ. In preparation, 2021.
[14] PsiQ. In preparation, 2021.

[15] Michael A. Nielsen. Quantum computation by measurement and quantum memory. *Physics Letters A*, 308(2-3):96–100, 2003.
[16] Daniel Gottesman and Isaac L. Chuang. Demonstrating the viability of universal quantum computation using teleportation and single-qubit operations. *Nature*, 402(6760):390–393, 1999.
[17] Frank Verstraete and J. Ignacio Cirac. Renormalization algorithms for quantum-many body systems in two and higher dimensions. *arXiv preprint cond-mat/0407066*, 2004.
[18] Robert Raussendorf and Hans J. Briegel. Computational model underlying the one-way quantum computer. *Quantum Info. Comput.*, 2(6):443486, October 2002.
[19] Debbie W. Leung. Quantum computation by measurements. *International Journal of Quantum Information*, 2(01):33–43, 2004.
[20] Daniel E. Browne and Terry Rudolph. Resource-efficient linear optical quantum computation. *Physical Review Letters*, 95(1):010501, 2005.
[21] PsiQ. In preparation, 2021.
[22] Alberto Politi, Jonathan C. F. Matthews, Mark G. Thompson, and Jeremy L. O'Brien. Integrated quantum photonics. *IEEE Journal of Selected Topics in Quantum Electronics*, 15(6):1673–1684, 2009.
[23] Warren P. Grice. Arbitrarily complete bell-state measurement using only linear optical elements. *Physical Review A*, 84(4):042331, 2011.
[24] Fabian Ewert and Peter van Loock. 3/4-efficient bell measurement with passive linear optics and unentangled ancillae. *Physical review letters*, 113(14):140403, 2014.
[25] Robert Raussendorf and Jim Harrington. Fault-tolerant quantum computation with high threshold in two dimensions. *Physical review letters*, 98(19):190504, 2007.
[26] Benjamin J. Brown and Sam Roberts. Universal fault-tolerant measurement-based quantum computation. *Physical Review Research*, 2(3):033305, 2020.
[27] Jean-Pierre Tillich and Gilles Zémor. Quantum ldpc codes with positive rate and minimum distance proportional to the square root of the blocklength. *IEEE Transactions on Information Theory*, 60(2):1193–1202, 2013.
[28] Daniel Gottesman. Fault-tolerant quantum computation with constant overhead. *arXiv preprint arXiv:1310.2984*, 2013.
[29] Dorit Aharonov and Michael Ben-Or. Fault-tolerant quantum computation with constant error rate. *SIAM Journal on Computing*, 2008.
[30] Eric Dennis, Alexei Kitaev, Andrew Landahl, and John Preskill. Topological quantum memory. *Journal of Mathematical Physics*, 43(9):4452–4505, 2002.
[31] Héctor Bombín. Single-shot fault-tolerant quantum error correction. *Physical Review X*, 5(3):031043, 2015.
[32] A. Bolt, G. Duclos-Cianci, D. Poulin, and T. M. Stace. Foliated quantum error-correcting codes. *Physical review letters*, 117(7):070501, 2016.
[33] Naomi Nickerson and Héctor Bombín. Measurement based fault tolerance beyond foliation. *arXiv preprint quant-ph/1810.09621*, 2018.
[34] Michael Newman, Leonardo Andreta de Castro, and Kenneth R Brown. Generating fault-tolerant cluster states from crystal structures. *Quantum*, 4:295, 2020.
[35] Eric Dennis, Alexei Kitaev, Andrew Landahl, and John Preskill. Topological quantum memory. *Journal of Mathematical Physics*, 43(9):44524505, Sep 2002.
[36] Nicolas Delfosse and Naomi H. Nickerson. Almost-linear time decoding algorithm for topological codes. *arXiv preprint arXiv:1709.06218*, 2017.
[37] Daniel Herr, Alexandru Paler, Simon J. Devitt, and Franco Nori. A local and scalable lattice renormalization method for ballistic quantum computation. *npj Quantum Information*, 4(1):1–8, 2018.
[38] James M. Auger, Hussain Anwar, Mercedes Gimeno-Segovia, Thomas M. Stace, and Dan E. Browne. Fault-tolerant quantum computation with nondeterministic entangling gates. *Physical Review A*, 97(3):030301, 2018.
[39] PsiQ. In preparation, 2021.
[40] H. J. Briegel, D. E. Browne, W. Dr, R. Raussendorf, and M. Van den Nest. Measurement-based quantum computation. *Nature Physics*, 5(1):1926, Jan 2009.
[41] Héctor Bombín and Miguel Angel Martin-Delgado. Quantum measurements and gates by code deformation. *Journal of Physics A: Mathematical and Theoretical*, 42(9):095302, 2009.
[42] Clare Horsman, Austin G. Fowler, Simon Devitt, and Rodney Van Meter. Surface code quantum computing by lattice surgery. *New Journal of Physics*, 14(12):123011, 2012.
[43] Héctor Bombín. Topological order with a twist: Ising anyons from an abelian model. *Physical review letters*, 105(3):030403, 2010.
[44] Paul Webster and Stephen D. Bartlett. Fault-tolerant quantum gates with defects in topological stabilizer codes. *Physical Review A*, 102(2):022403, 2020.
[45] S. B. Bravyi and A. Y. Kitaev. Quantum codes on a lattice with boundary, arxiv. *arXiv preprint quant-ph/9811052*, 1998.
[46] Christopher Chamberland, Pavithran Iyer, and David Poulin. Fault-tolerant quantum computing in the pauli or clifford frame with slow error diagnostics. *Quantum*, 2:43, Jan 2018.
[47] L. Riesebos, X. Fu, S. Varsamopoulos, C. G. Almudever, and K. Bertels. Pauli frames for quantum computer architectures. In *Proceedings of the 54th Annual Design Automation Conference 2017*, DAC '17, New York, NY, USA, 2017. Association for Computing Machinery.
[48] Daniel Litinski. A game of surface codes: Large-scale quantum computing with lattice surgery. *Quantum*, 3:128, 2019.
[49] Vladimir Kolmogorov. Blossom v: a new implementation of a minimum cost perfect matching algorithm. *Mathematical Programming Computation*, 1(1):43–67, 2009.
